# Bi-allelic loss-of-function variants in *POC5* cause a syndromic retinal, endocrine, and neuromuscular ciliopathy

**DOI:** 10.1016/j.gim.2025.101513

**Published:** 2025-06-28

**Authors:** Anneke T. Vulto-van Silfhout, Ingrid M. Jazet, Suzanne Yzer, Jeroen Pas, Serwet Demirdas, Elisabeth F.C. van Rossum, Alberta A.H.J. Thiadens, Ronald van Beek, Lonneke Haer-Wigman, Daniela Q.C.M. Barge-Schaapveld, Charlotte Brasch-Andersen, Simon Frost, Miriam Bauwens, Elfride De Baere, Irina Balikova, Filip Van den Broeck, Monika Weisz-Hubshman, Pascal Joset, Peter Miny, Isabel Filges, Susanne Kohl, Pietro De Angeli, Laura Kühlewein, Jan-Philipp Bodenbender, Tobias Haack, Karin Poths, Lidia Fernandez-Caballero, Marta Corton, Fiona Blanco Kelly, Carmen Ayuso, Peggy Martínez-Esteban, John Vissing, Jordi Díaz-Manera, Volker Straub, Ana Töpf, Siying Lin, Gavin Arno, William L. Macken, Jennifer Spillane, Radha Ramachandran, Erik de Vrieze, Tjakko van Ham, Susanne Roosing, Machteld M. Oud

**Affiliations:** 1 Department of Human Genetics, Radboud University Medical Center, Nijmegen, The Netherlands; 2 Department of Clinical Genetics, Maastricht University Medical Center, Maastricht, The Netherlands; 3 Division of Endocrinology, Department of Medicine, Leiden University Medical Center, Leiden, The Netherlands; 4 Department of Ophthalmology, Radboud University Medical Center, Nijmegen, The Netherlands; 5 Department of Clinical Genetics, Erasmus University Medical Center, Rotterdam, The Netherlands; 6 Division of Endocrinology, Department of Internal Medicine, Erasmus University Medical Center, Rotterdam, The Netherlands; 7 Department of Ophthalmology, Erasmus University Medical Center, Rotterdam, The Netherlands; 8 Department of Clinical Genetics, Leiden University Medical Center, Leiden, The Netherlands; 9 Department of Clinical Genetics, Odense University Hospital, Odense, Denmark;; 10 Center for Medical Genetics, Ghent University and Ghent University Hospital, Ghent, Belgium; 11 Department of Ophthalmology, University Hospital Leuven, Leuven, Belgium; 12 Department of Ophthalmology, Ghent University Hospital, Ghent, Belgium; 13 Department of Molecular and Human Genetics, Baylor College of Medicine, Houston, TX; 14 Texas Children’s Hospital, Houston, TX; 15 Medical Genetics, Institute of Medical Genetics and Pathology, University Hospital Basel, Basel, Switzerland; 16 Molecular Genetics Laboratory, Institute for Ophthalmic Research, Center for Ophthalmology, University, Tübingen, Germany; 17 University Eye Hospital, Center for Ophthalmology, Eberhard Karls University, Tübingen, Germany; 18 Institute for Medical Genetics and Applied Genomics, Eberhard Karls University, Tübingen, Germany; 19 Department of Genetics & Genomics, Instituto de Investigación Sanitaria-Fundación Jiménez Díaz University Hospital, Universidad Autónoma de Madrid (IIS-FJD, UAM), Madrid, Spain; 20 Center for Biomedical Network Research on Rare Diseases (CIBERER), Instituto de Salud Carlos III, Madrid, Spain; 21 Neurofisiologia Clínica, Instituto Nacional de Salud del Niño San Borja, Lima, Perú; 22 Copenhagen Neuromuscular Center, Rigshospitalet, University of Copenhagen, Copenhagen, Denmark; 23 The John Walton Muscular Dystrophy Research Centre, Translational and Clinical Research Institute, Newcastle University and Newcastle Hospitals NHS Foundation Trust, Newcastle upon Tyne, United Kingdom; 24 NIHR Biomedical Research Centre, Moorfields Eye Hospital and the UCL Institute of Ophthalmology, London, United Kingdom; 25 UCL Institute of Ophthalmology, University College London, London, United Kingdom; 26 Division of Research, Greenwood Genetic Center, Greenwood, South CA; 27 Department of Neuromuscular Diseases, UCL Queen Square Institute of Neurology, London, United Kingdom; 28 NHS Highly Specialised Service for Rare Mitochondrial Disorders, Queen Square Centre for Neuromuscular Diseases, The National Hospital for Neurology and Neurosurgery, London, United Kingdom; 29 Department of Adult Inherited Metabolic Diseases, Metabolic Medicine and Chemical Pathology, Guys and St Thomas’ Hospitals NHS Foundation Trust, London, United Kingdom; 30 Department of Otorhinolaryngology, Radboud University Medical Center, Nijmegen, The Netherlands

**Keywords:** Ciliopathy, Insulin resistance, Muscle cramps, Renal disease, Syndrome

## Abstract

**Purpose:**

A homozygous loss-of-function (LoF) variant in *POC5* was previously described in an individual with retinitis pigmentosa. We identified *POC5* variants in 12 probands with a syndromic phenotype. We aim to define the phenotype spectrum and molecular mechanism associated with biallelic *POC5* LoF variants.

**Methods:**

We studied a cohort of 12 families with bi-allelic LoF *POC5* variants and performed detailed phenotype analysis. POC5 localization studies were performed in 3 proband-derived fibroblast cell lines.

**Results:**

Detailed phenotyping of probands with *POC5* variants expands the phenotype spectrum beyond ocular manifestations. This syndrome causes not only rod-cone dystrophy but also diabetes mellitus with severe insulin resistance and partial lipodystrophy, kidney disease, and muscle cramps. The POC5 protein plays an essential role during cell cycle and cilium formation. Interestingly, POC5 localization studies in 3 proband-derived fibroblast cell lines show aberrant localization suggesting a ciliary defect. The phenotypes of the 12 families in this study fit well within the ciliopathy phenotype spectrum, except for lipodystrophy, which is not common in ciliopathies.

**Conclusion:**

We describe a multiorgan syndrome caused by bi-allelic LoF variants in *POC5*. This underscores the pleiotropic effects of *POC5* variants and highlights the significance of adipose tissue and metabolic dysfunction in ciliopathies.

## Introduction

A homozygous loss-of-function (LoF) variant in *POC5 centriolar protein (POC5*, HGNC:26658) was initially described in an individual with retinitis pigmentosa (RP).^1^ RP encompasses a heterogeneous group of inherited retinal disorders characterized by progressive degeneration of photoreceptor cells, leading to night blindness, peripheral visual field loss, and eventual blindness. Variants in numerous genes with variable functions have been implicated in RP.^2,3^ An important mechanism underlying the development of RP is disruption of ciliary function. Several genes encoding ciliary and centrosomal proteins, such as *RPGR* (HGNC:10295), *RPGRIP1* (HGNC:13436), and *CEP290* (HGNC:29021), have been identified as causative factors in RP.^4^ Cilia are microtubule-based organelles protruding from the cell surface, critical for cellular signaling and sensory functions. Interestingly, POC5 is a conserved centriolar protein that is also present in the basal body of primary cilia.^5^

Perturbations in ciliary proteins have been associated with a spectrum of ciliopathies. Ciliopathies are a clinically heterogeneous group of disorders that can affect almost any organ of the human body. This can result in diverse clinical phenotypes often encompassing retinal degeneration, renal anomalies, obesity, and skeletal abnormalities.^6^

The single-case report by Weisz Hubshman and colleagues focused on the RP phenotype. However, it was noted that this individual also had short stature, microcephaly, renal problems due to recurrent glomerulonephritis, and episodic muscle pain with elevated serum creatine kinase (CK) levels.^1^ In addition, other studies have linked pathogenic missense *POC5* variants to idiopathic scoliosis,^7,8^ through disturbance of the ciliary and centrosomal role of this protein.^9^ This suggests a more pleiotropic effect of *POC5* variants on other organ systems beyond ocular manifestations.

We describe a multiorgan syndrome caused by biallelic LoF variants in *POC5*. The main features consist of rodcone dystrophy (RCD), diabetes mellitus with high insulin resistance, lipodystrophy (LD), muscle cramps, and renal failure, expanding the phenotypic spectrum associated with POC5 dysfunction. Moreover, we show aberrant localization of POC5 at the basal body of the cilium, providing evidence that the described syndrome is a ciliopathy.

## Materials and Methods

### Inclusion of participants

Probands with biallelic pathogenic *POC5* variants were collected at the Radboud University Medical Center, Nijmegen. These individuals were obtained via 11 medical centers from all over the world, through GeneMatcher (participant (P)2, P9), the European Retinal Disease consortium (www.erdc.info) (P7, P11, and P12), and other research collaborations.^10^ Additional phenotype data were collected on the previously published proband of Weisz Hubshman and colleagues.^1^ Informed consent was obtained from all participants. This study adhered to the World Health Association Declaration of Helsinki (2013) and was approved by the local ethics committee of the Radboud University Medical Center (Nijmegen, The Netherlands), the Rotterdam Eye Hospital (Rotterdam, The Netherlands) (MEC-2010–359; OZR protocol no. 2009–32), the Fundación Jiménez Díaz Hospital Research (Approval No.: PIC172–20_FJD), the Ethics Board of the Medical Faculty of the University of Tübingen (project no. 116/2015BO2 and 124/2015BO1), and the National Research Ethics Service (NRES) Committee North East-Newcastle and North Tyneside 1 (19/NE/0028).

### Collection of phenotype data

Detailed phenotype data were collected retrospectively. A standardized questionnaire was sent to the involved medical specialists. Detailed clinical ophthalmologic, endocrinologic, and systemic data were obtained if available. Photographs of participants were collected upon written informed consent for publication.

### Genetic analysis

Genetic analysis of the probands was performed through exome or genome sequencing by established procedures (see Supplemental descriptions). *POC5* variants were identified through diagnostic or research analysis. Variants in *POC5* (NM_001099271.2) are reported according to Human Genome Variation Society variant nomenclature.^11^ All variants were submit to the Leiden Open Variant Database. Segregation analysis of the identified *POC5* variants was performed using Sanger sequencing or exome sequencing on available family members.

### Targeted and transcriptome-wide RNA analysis

Targeted RNA analysis was performed on RNA from fibroblasts from P3, P8, P9, P10, and 2 unrelated controls. The fibroblasts were cultured in the presence or absence of cycloheximide (CHX). A nearly full-length POC5 transcript was amplified with polymerase chain reaction (PCR) using a forward primer located in exon 2 and a reverse primer in exon 12 (see Supplemental Table 1 for primer sequences). Transcriptome-wide analysis was performed for P3 as described by Dekker et al.^12^ In brief, RNA was isolated from fibroblasts cultured with and without CHX. Expression outliers were picked-up by a transcriptome-analysis web application (established by Department of Clinical Genetics Erasmus MC, Rotterdam, The Netherlands) identifying abnormal transcripts in one data set compared with a control cohort using exon, intron, and gene-level outliers based on the OUTRIDER algorithm.^13^ Protein notation of a *POC5* variant was modified following HGVS rules in light of the outcome of RNA studies.

### Quantitative real-time polymerase chain reaction

Quantitative real-time PCR was performed on RNA from fibroblasts from P3, P8, P9, P10, and 3 unrelated controls. The relative expression levels of *POC5* were determined by quantitative real-time PCR using GoTaq qPCR Master mix (Promega). The results were analyzed using the Delta-delta-Ct method, using *GUSB* as a housekeeping gene for normalization. The primers used are listed in the Supplemental Table 2.

### Immunofluorescence

Skin-derived fibroblasts from P3, P8, P9, and P10 were cultured for immunofluorescent analysis of POC5, centrin, ciliogenesis, cilium length, and sonic hedgehog (SHH) signaling. The fibroblasts were serum starved for 48 hours before fixation to stimulate cilium formation. For the SHH assay, cells were stimulated with 500 nM smoothened agonist (or dimethyl sulfoxide as negative control) for 24 hours to activate the SHH pathway. In brief, upon fixation, the cells were stained with antibodies targeting POC5 (rabbit polyclonal, 1:250), ARL13B (rabbit polyclonal, 1:100), acetylated-α-tubulin (mouse monoclonal, 1:1000), GT335 (mouse monoclonal, 1:1000), centrin (mouse monoclonal, 1:1000), pericentrin (mouse monoclonal, 1:1000), smoothened (mouse monoclonal, 1:500), and GPR161 (rabbit polyclonal, 1:1000). Subsequently, the coverslips were microscopically analyzed for POC5 and centrin localization and the ciliary phenotype, including ciliogenesis, cilium length, and SHH, according to the ALPACA method as described by Doornbos et al.^14^ The experiments were performed in triplicate.

## Results

### Identification of POC5 variants

**Figure 1:**
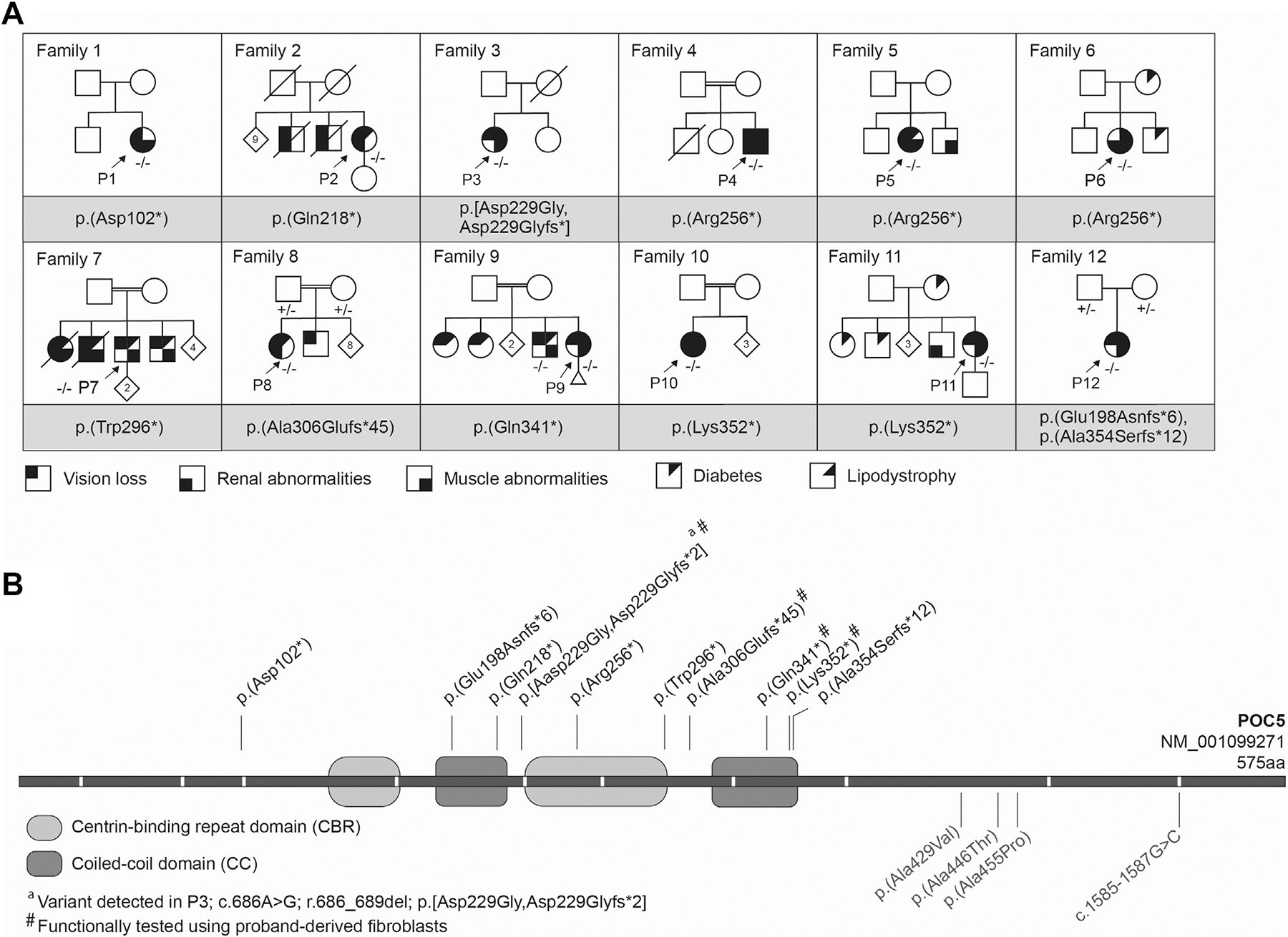
Twelve families with *POC5* LoF variants. A. Pedigrees of the 12 families. Shaded symbols indicate the presence of various phenotype features in probands and their family members. Results of genetic analysis are indicated (− = *POC5* variant). B. Schematic representation of the POC5 protein structure with its predicted functional domains. The white lines in the bar indicate the exon-exon junctions. The variants above the bar in black are LoF variants described in this study, and variants below the bar in gray have previously been associated with adolescent idiopathic scoliosis.^7^

Twelve families from various ethnic backgrounds with biallelic LoF variants in *POC5* were identified (Figure 1, Table 1). Eleven probands were diagnosed with homozygous *POC5* variants, whereas compound heterozygous *POC5* variants were identified in family 12. Ten different *POC5* variants were identified, with 2 variants found in multiple unrelated families, each from different ethnic and geographical origins.

*POC5* is considered tolerant to LoF in the general population; however, no homozygous LoF variants were present in the Genome Aggregation Database (gnomAD v4.1).^15^ The maximal frequency of a heterozygous *POC5* LoF variant (excluding low-confidence calls) was 28/1602202 alleles resulting in an allele frequency of 1.75e^−5^. The heterozygous frequencies of all identified variants in these studies are provided in Supplemental Table 3.

### RNA analysis of POC5 variants showed reduced expression

**Figure 2:**
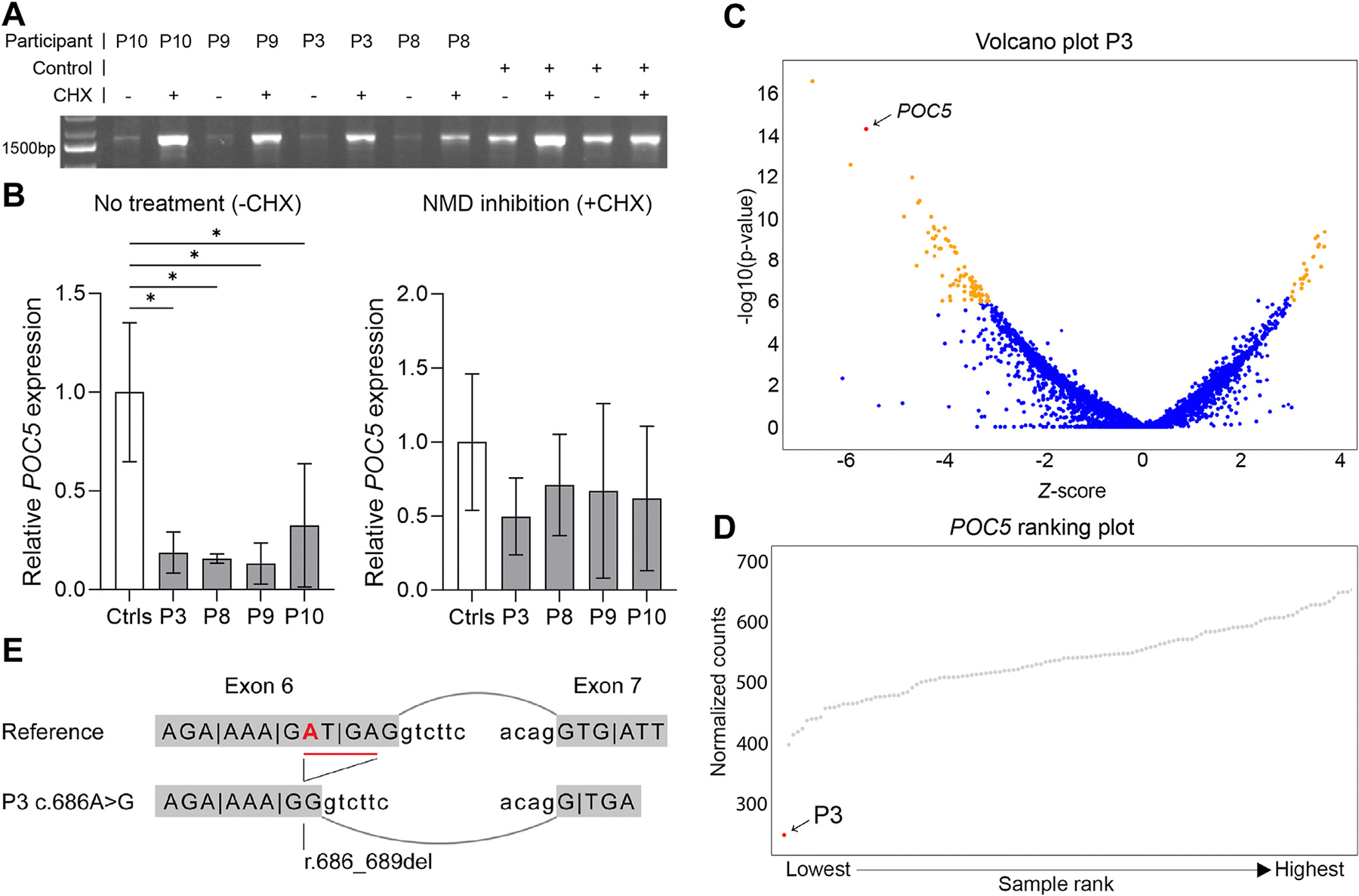
RNA and protein analysis of *POC5*-affected participants. A. Targeted RNA analysis of *POC5* in fibroblasts treated with (+) or without (−) CHX. B. Quantitative RNA analysis of *POC5* in fibroblasts treated with (+) or without (−) CHX to inhibit NMD. Bars represent the mean with SD of *POC5* expression relative to the controls (data from 2 or 3 replicates per fibroblast line). Statistical significance was calculated using analysis of variance with post-hoc multiple comparison using Dunnett’s test (* is *P* < .05). C and D. Reduced expression of POC5 transcript caused by homozygous variant c.686A>G in *POC5* that leads to a 4-bp deletion (r.686_689del) in the POC5 transcript. C. Volcano plot of P3 showing expression outlier genes in orange with *z*-score >3 and *P* value < .01. POC5 has a *P* value of 5.9e^−07^ and a *z*-score of −5.60. D. Gene ranking plot for POC5 showing P3 in red with a normalized count of 250 and unrelated control samples in gray with a mean of 544. E. Schematic visualization of the splice effect caused by variant c.686A>G seen in P3. CHX, cycloheximide; NMD, nonsense-mediated messenger RNA (mRNA) decay.

Six out of 10 identified variants were nonsense variants, whereas 4 variants introduced a frameshift, all resulting in a premature termination codon. All variants were expected to result in nonsense-mediated messenger RNA (mRNA) decay and therefore a loss of protein. To check this hypothesis, RNA studies were performed on fibroblasts, with or without CHX, from participants 3, 8, 9, and 10. Targeted analysis on cDNA showed a specific band for *POC5* in all samples. The expression level of *POC5* in all 4 participants is significantly reduced (Figure 2A and B). Upon treatment with CHX, the participant samples showed a clear increase in the amount of RNA expression (Figure 2A and B), suggesting that the majority of *POC5* transcript is subjected to nonsense-mediated mRNA decay.

For P3, the detection of the causative *POC5* variant started with a transcriptome-wide analysis. Transcriptome expression outlier analysis on RNA from P3 showed strongly reduced expression of *POC5* (*z*-score −5.60 with a *P* value of 5.9e^−7^) in the participant compared with unrelated controls (Figure 2C and D, Supplemental Figure 1). With this knowledge, a putative missense variant Chr5(GRCh38):g.75694659T>C c.686A>G p.(Asp229Gly) was detected in the DNA of P3. This variant is located 5 nucleotides upstream of the exon-intron boundary of exon 6 in the coding region, and SpliceAI predicted the creation of a novel splice donor site (SpliceAI Δ score 0.99). The expressed *POC5* transcript showed a 4 base-pair deletion at mRNA level r.686_689del (NM_001099271.2), providing evidence that c.686A>G causes abnormal splicing through activation of a novel splice donor site, leading to a frameshift p.[Asp229Glyfs*2,Asp229Gly] and a premature termination codon (Figure 2E).

### Detailed phenotype analysis

**Figure 3:**
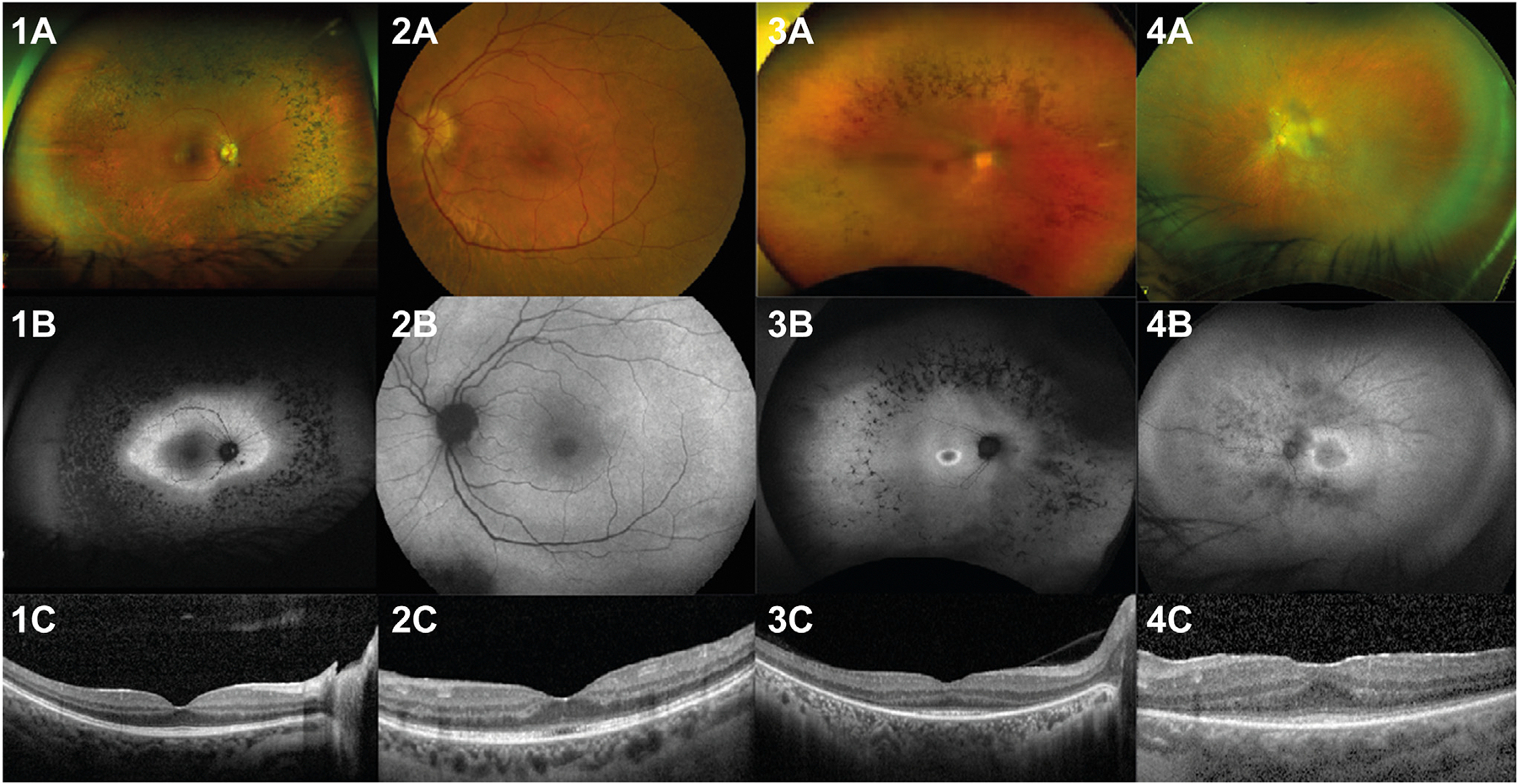
Representative ophthalmologic images of *POC5* participants. (1) Ophthalmologic imaging of RE of P10 (22-year-old female) with (A) wide-field fundus photograph showing peripheral bone-spicule hyperpigmentation, (B) wide-field green-light fundus autofluorescence image showing a clear hyperautofluorescent ring surrounding the central retina and, further peripherally, a ring of hypo-autofluorescent spots, (C) optical coherence tomography (OCT) image with preserved ellipsoid zone. (2) Ophthalmologic imaging of LE of P11 (54-year-old female) with (A) fundus photograph showing a waxy pallor visible on the optic nerve head, (B) blue-light fundus autofluorescence image is essentially normal centrally and shows a hypo-autofluorescent area inferonasally to the arcade, (C) subfoveal disruption of ellipsoid zone on OCT. (3) and (4) present P8 RE and P5 LE, respectively, with a similar ocular phenotype as P10. LE, left eye; RE, right eye.

**Figure 4:**
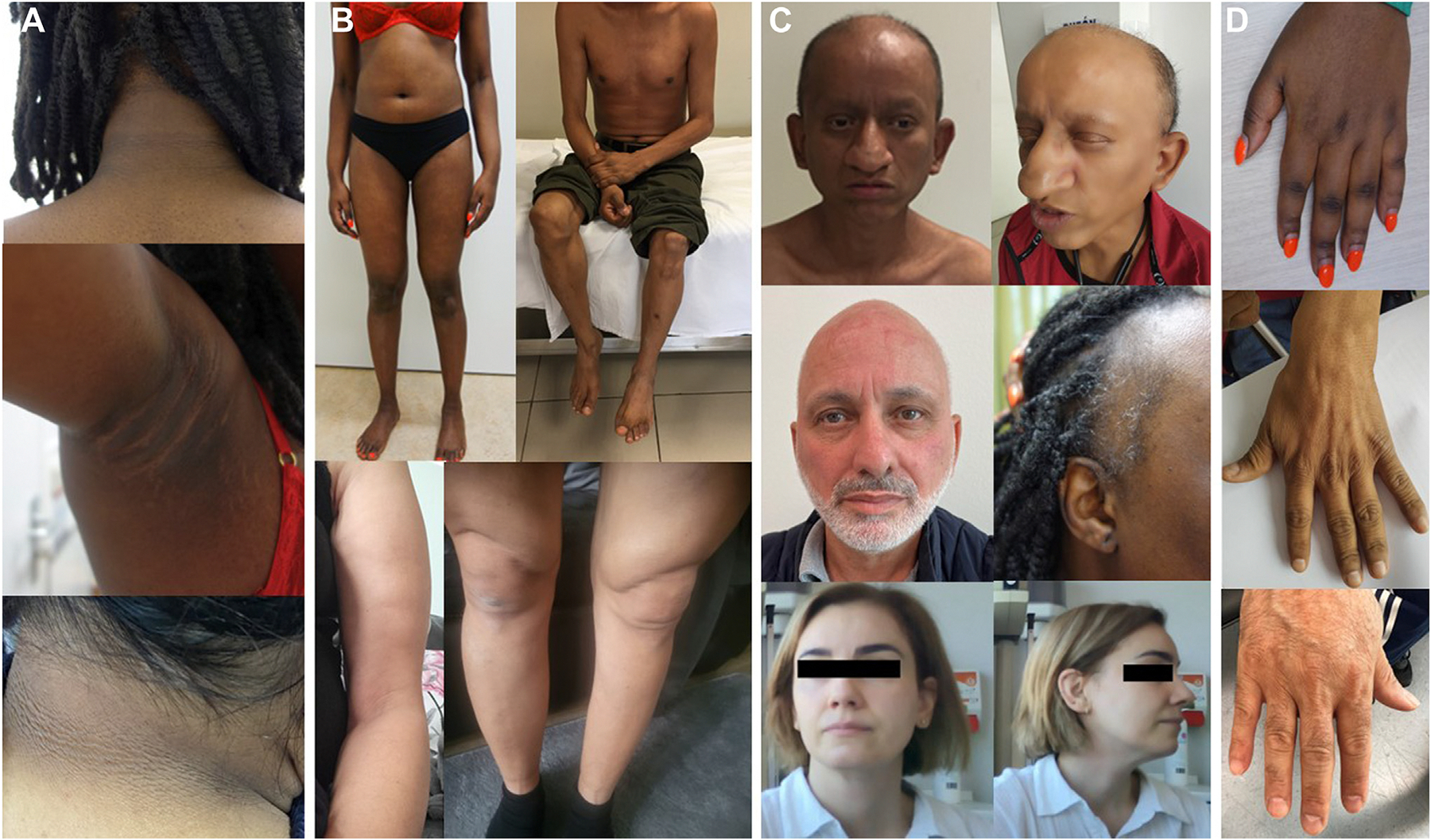
Photographs of participants. A. Acanthosis nigricans in neck of P3, acanthosis nigricans and striae in axilla of P3, and acanthosis nigricans in neck of P8. B. Full body photograph of P3 (26-year-old female) showing slim build with decreased subcutaneous fat and subtle central adiposity, full body photograph of P4 (46-year-old male) showing muscular appearance with decrease of subcutaneous fat, and arm and leg of P6 (48-year-old female) showing asymmetric fat distribution in her thighs and reduced subcutaneous fat in the upper arms. C. Frontal facial photographs of P4, P7, and P12 and lateral of P4, P3, and P12 showing variable dysmorphic features, including wide nasal base with thick nasal alae and a low-hanging columella, mandibular prognathia, and large ears. D. Photographs of hands of P3, P4, and P7 showing brachydactyly and loss of subcutaneous fat in P4.

Detailed phenotype information could be obtained from 12 probands with biallelic *POC5* variants (Figures 1, 3, and 4, Table 1, Supplemental Table 3, and Supplemental descriptions). P1 was published previously.^1^ The participants were 19 to 59 years of age (median age 37 years). Two were males, and 10 were females.

### Retinal dystrophy and other abnormalities of the eye

All participants except 1 (P6) showed a RCD (Figure 3). The age of onset of ocular symptoms was mostly in the teenage years. Best corrected visual acuity was variable; however, most cases had preserved central vision of 20/20 Snellen until their thirties. Refraction varied from +3.5 to −7.5 spherical equivalent in the nonpseudophakic individuals.

Slit lamp examination of the anterior segment showed mild cataract or pseudophakia in 5 participants. On dilated fundus examination, the macula showed mild granular changes of the retinal pigment epithelium (RPE) in most cases. There was attenuation of the retinal vasculature. In the midperipheral retina, just outside the vascular arcades, an atrophic retina and, adjacent (more peripheral) to this, a bone-spicule hyperpigmentation, was visible. The far periphery was atrophic.

Electroretinography showed severely reduced amplitudes to extinguished scotopic and photopic responses. When still detectable, scotopic responses were affected more severely compared with photopic responses. Visual field testing showed severe concentric constriction in all cases that underwent this test.

Multimodal imaging was performed in 7 cases and showed preserved outer retina within the vascular arcades with normal configuration of the fovea on optical coherence tomography, except for P11, who showed an abnormal outer retina in the fovea of both eyes. Fundus autofluorescence showed normal posterior poles with a hyperautofluorescent ring visible around the arcades and nasal to the optic disc in a 22-year-old case (P8).

P3 deviated from the ocular phenotype described above because poor vision and roving eye movements were noted in early childhood. Best corrected visual acuity at age 9 was counting fingers in both eyes and slit lamp examination revealed both horizontal and vertical nystagmus. Electroretinography showed residual cone function (10% of expected amplitudes) without detectable rod function. Visual field testing showed a tunnel vision limited to the central 10 degrees. She was subsequently diagnosed with an early-onset RCD.

### Abnormalities of the endocrine system

Ten participants were diagnosed with diabetes mellitus at an early age (median age: 26 years, age range: 13–49 years). This was characterized by high insulin resistance in 8 participants, which was reflected by high insulin levels (eg, insulin of 1076 pmol/L [reference range 12–96 pmol/L] in P3), acanthosis nigricans in the axillary and inguinal regions, as well as in other areas in P3, P8, P9, and P10 (Figure 4A) and hepatic steatosis in P3–6, P8–12.

Interestingly, 6 participants (P3, P4, P6, P9, P10, and P12) showed signs of LD. They had a loss of subcutaneous fat from the upper and lower limbs, leading to a slim and muscular build (Figure 4B), whereas accumulation of subcutaneous fat in the abdomen was observed. This was most striking in P10, who showed a steatosis hepatis since the age of 8 years and was diagnosed with diabetes mellitus with high insulin resistance at the age of 15 years. She had clear loss of subcutaneous fat in the face and from the upper and lower extremities and the diagnosis of partial lipodystrophy was established by a lipodystrophy expert center. The LD in the 6 participants was noticeable since childhood or puberty. Their body mass index (BMI) was low (median 19 kg/m^2^; range 16–24 kg/m^2^). In these 6 participants, but also in P5, P8, and P11, hepatic steatosis with elevated liver enzymes and/or an abnormal lipid profile was observed. In P11, a liver biopsy showed mild steatohepatitis, as well as mild periportal and centrilobular fibrosis. Highly elevated triglycerides were observed in P5 and P6. Unexpectedly, P5 died because of acute pancreatitis at the age of 32 years.

The diabetes mellitus in the participants was initially treated with metformin. This had a good effect in P3, P5, and P7 with a decrease in serum insulin levels and reversal of liver steatosis in P3. The other participants required additional treatment with sulfonylurea derivatives, dipeptidyl peptidase-4 inhibitors and insulin. However, in P10, diabetes remained poorly controlled and because of low serum leptin levels (5.1 μg/L; reference >12 μg/L), recombinant human metraleptin therapy was started with good effect on glucose regulation, hepatic steatosis, and albuminuria.

Problems of the reproductive system were reported in 9 participants, including secondary oligo-/amenorrhea and irregular menstruation, ovarian cysts, and polycystic ovary syndrome. Other signs of hyperandrogenism, such as hirsutism, acne, and increased serum testosterone levels, were also observed in the females. One of the male participants showed a low testosterone level. Three participants (1 male and 2 females) had children.

### Additional phenotype features

Ten participants reported neuromuscular abnormalities. Eight participants (P1, P4–7, and P9–12) had intermittent, involuntary painful muscle cramps since childhood. Muscle weakness, fasciculations, dystonia, and abnormalities on electromyography were also reported. In P1 and P5–7, serum CK levels were reported to be elevated, with a suspicion of rhabdomyolysis in P5. Muscle MRI in P5 did not demonstrate signs of a myopathy. Four participants underwent muscle biopsy. Two showed nonspecific myopathic changes (mild fiber size variation due to preferential fast fiber atrophy, single necrotic fiber, and widespread unevenness of oxidative staining in both fiber types in P5, type 2 fiber atrophy in P6, some muscle necroses, and many central nuclei in P7), whereas results were normal in P12. Muscle cramps were often disabling and difficult to treat, and only P5 showed a positive response to Quinine with a reduction in frequency of muscle cramps from weekly to monthly.

Four participants (P2, P4, P6, and P8) had renal insufficiency, with 1 having undergone a kidney transplantation at the age of 37 years (P2). It is striking that both P2 and P6 had 2 siblings who died of renal failure at an early age (midthirty, 42, 64, and 66 years, respectively). However, segregation analysis to confirm *POC5*-associated disease could not be performed because DNA was unavailable. P5 had a single functional kidney since birth, and P9 showed albuminuria.

In all participants, stature was relatively short (median −1 SD on WHO growth charts, range −3.4 to +0.1 SD). Weight varied from −2.2 to +1.9 SD (BMI 16 to 27 kg/m^2^). Head circumference was only available for 3 participants, 1 had a severe microcephaly (−3.8 SD; P1), whereas the other 2 had relatively small head circumference (both −1.6 SD). Dysmorphic features were observed in 5 participants, mainly consisting of a wide nasal base with thick nasal alae and a low-hanging columella, mandibular prognathia, and large ears (Figure 4C). Sparse hair or alopecia was observed in 6 participants (P3–5, P8, P10, and P12), including also sparse eyebrows and eyelashes. P4 also had conical teeth and a thick skin and was diagnosed with an ectodermal dysplasia. Other skin abnormalities consisted of soft skin (P1, P10), with easy/atypical scarring (P5, P10) and striae (P3, Figure 4A). In individuals P4 and P7, brachydactyly was observed, whereas P4–6 and P10 had large hands and feet (Figure 4D). Scoliosis was observed in 2 participants (P3, P12). Other skeletal problems included osteoarthrosis and degenerative back pain in P11, unspecified joint and orthopedic complications in P2, and hip dysplasia and pectus excavatum in P10. In P5 and P6, a mild cardiac hypertrophy was observed. Motor and cognitive development were normal in all participants.

### Family history

Consanguinity was reported in 5 families. The other families were not known to be related, but in 3 families, the parents originated from a small community that may be enriched for homozygous variants due to endogamy.

In 6 families, siblings showed phenotype abnormalities that could also be consistent with a *POC5*-related disorder (Figure 1). A brother of P8 was reported to be affected with RCD, diabetes, and muscle cramps. Segregation analysis showed he was also homozygous for the *POC5* variant. They also had 2 sisters with blindness and diabetes, in whom genetic testing could not be performed.

In the other families, siblings were not available for genetic testing. P2 had 2 brothers who also suffered from vision impairment and died of renal failure. P6 had 3 affected siblings all with severe muscle cramps with elevated CK, RCD (2 of them completely blind) and diabetes. Two of the affected were deceased at age 64 and 66, allegedly because of kidney failure. P7 had a brother with visual impairment of whom no further information was available.

### POC5 mislocalization in fibroblasts derived from participants

**Figure 5:**
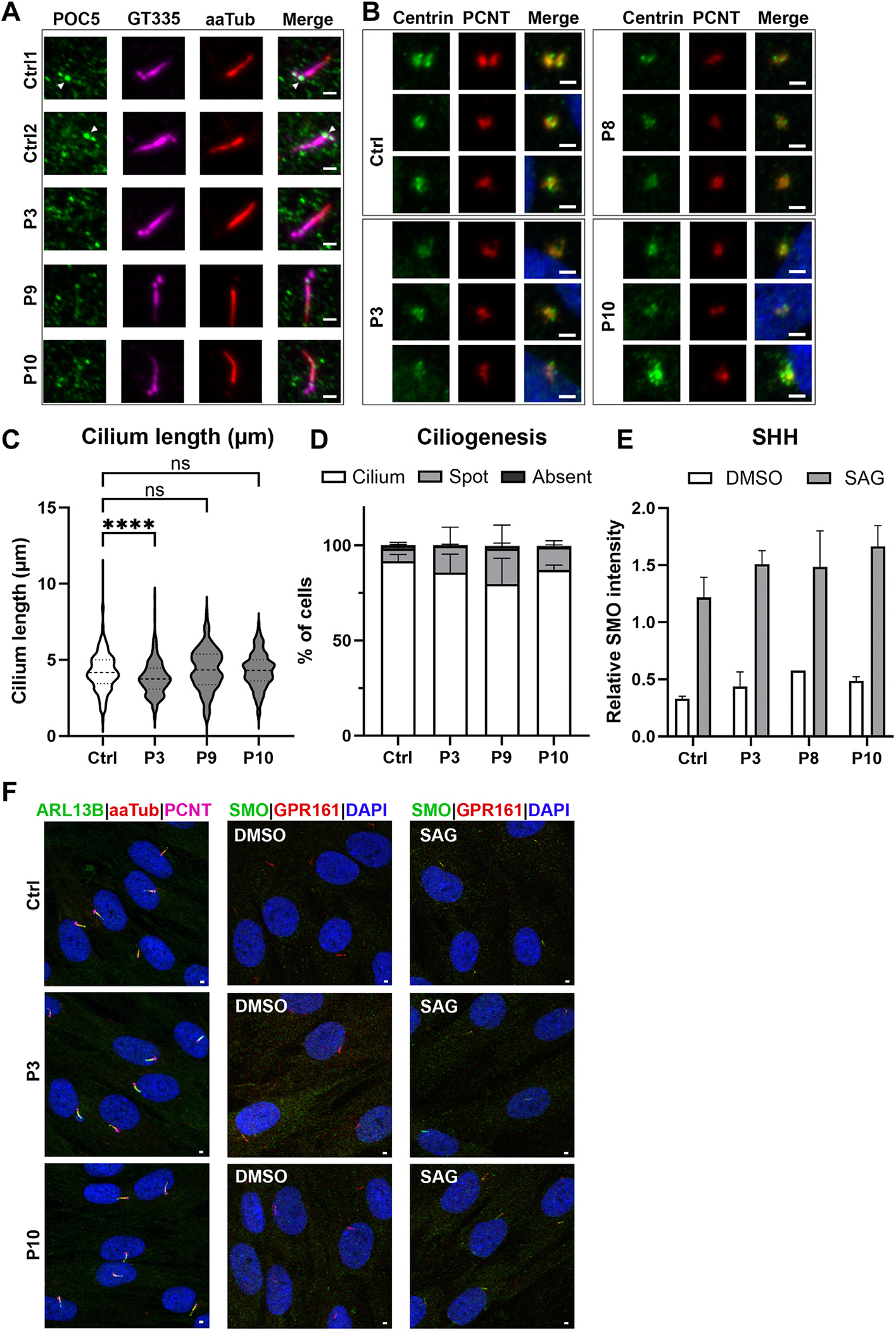
Immunofluorescent imaging of POC5, centrin and cilium phenotype markers in fibroblasts from controls and P3, P8, P9, and P10. A. Centriolar localization of POC5 in ciliated cells. The images are representative of the analysis based on 75 cells per cell line that was performed in triplicate. Centrioles and the proximal end of the cilium are visualized with GT335 (purple), the ciliary axoneme is stained with anti-acetylated-α-tubulin (red) and POC5 localization is visualized in green. The mother centriole or basal body is indicated with a white arrowhead and the scale bar represents 1 μm. B. Centriolar localization of centrin in ciliated cells. The images are representative of a *n* = 2 analysis based on 75 cells per cell line. Centrin is visualized in green, and pericentrin is visualized in red. C and D. No substantial differences were observed in cilium length (C) or ciliogenesis (D) between controls and affected participants. The cilium length was based on the combined signal of acetylated-α-tubulin and ARL13B and was measured in an automated manner as described by Doornbos et al.^13^ Cilia from affected P3 were only mildly shorter compared with controls (combined data from 2 control cell lines) (ie, 3.8 ± 0.2 and 4.19 ± 0.17 μm, respectively). Statistical significance was calculated using analysis of variance with post-hoc multiple comparison using Dunnett’s test (**** is *P* < .0001). E. Quantification of SHH assay showing the relative fluorescent intensity signal of SMO over GPR161 in the cilium. Fibroblasts were stimulated with SAG or DMSO as a negative control. F. Representative cilia images upon 48 hours of serum starvation with or without SAG stimulation. Panel 1 was used for ciliogenesis and cilium length measurements and panel 2 and 3 for the SHH assay. Staining for panel 1 includes anti-ARL13B (green), anti-acetylated-α-tubulin (red), and anti-PCNT (purple). Staining for panels 2 and 3 includes anti-SMO (green) and anti-GRP161 (red). Scale bar represents 1 μm. DMSO, dimethyl sulfoxide; PCNT, pericentrin; SAG, smoothened agonist; SHH, sonic hedgehog; SMO, smoothened.

Because POC5 is important for cilia formation, we investigated primary cilia, and the localization of POC5 and centrin using immunofluorescence on skin-derived fibroblasts from P3, P8, P9, and P10. In controls, POC5 localizes to both GT335-positive centrioles that together form the centrosome (Figure 5A). Upon serum starvation, cilium formation was induced from the mother centriole, forming the basal body. Fibroblasts from P3, P9, and P10 did not show a clear POC5 signal at either of the 2 centrioles (Figure 5A, Supplemental Figure 2). Because POC5 is a centrin-binding protein we investigated the localization of centrin and showed a diffuse centriolar signal in cells from participants P3, P8, and P10 compared with the expected 2 foci signal observed in controls (Figure 5B).

Because POC5 localizes to the basal body of primary cilia, we studied whether the ability to form cilia and/or the cilium length were altered in cells from the participants. On average 92% of the control cells were ciliated, and this was similar in the participants’ cells, ie, 85%, 80%, and 87% for P3, P9, and P10, respectively. Cilium length measurements showed no significant difference when comparing those of control cells (4.19 ± 0.17 μm) with those of cells from P9 and P10 (4.36 ± 0.11 and 4.28 ± 0.29 μm, respectively). Interestingly, P3 showed mild but significantly shorter cilia (ie, 3.80 ± 0.20 μm) compared with the other cell lines (Figure 5C-E, Supplemental Table 4). To determine whether the functionality of cilia was affected in absence of POC5, we studied the ciliary SHH signaling pathway. During activation of the SHH pathway, GPR161 is transported out of the cilium, and smoothened (SMO) is transported into the cilium. The signal switch between GPR161 and SMO in the cilium upon SHH activation was clearly detected in all evaluated fibroblast lines (ctrl, P3, P8, and P10). To objectively determine whether the pathway was on or off, the fluorescent signal ratio was measured between SMO and GPR161 per cilium, and this determined no difference between the controls and the participants (Figure 5E and F).

## Discussion

This study shows that biallelic *POC5* LoF variants cause a multiorgan syndrome. This not only includes an RCD but also encompasses among others diabetes mellitus with insulin resistance, signs of partial LD, kidney failure, and muscle cramps. Participants were often undiagnosed until the appearance of their RCD, whereas they presented with other symptoms previously. Therefore, we recommend *POC5* testing also in individuals with early-onset diabetes type 2, signs of LD, muscle cramps, and/or abnormalities of the kidney.

The fact that 11 participants are insulin resistant and have hepatic steatosis, along with at least skinny legs, abdominal fat deposits, and a low to low-normal BMI in 6 participants, suggests an association with partial LD.^16^ LD is characterized by abnormal fat distribution and adipose tissue dysfunction leading to metabolic disturbances that are severe and therapy resistant.^17^ Because many participants in our study were diagnosed because of visual impairment, the slim and muscular build can be easily overlooked by an ophthalmologist.^18^ It is interesting to note that, additionally, for the previously reported P1, a thin habitus was observed that was previously unreported.^1^

The presence of diabetes mellitus with insulin resistance and LD can explain several of the organ abnormalities that were observed in our participants, such as the hepatic steatosis, hyperandrogenism, albuminuria, and glomerulonephritis.^17^ Additionally, muscle problems and elevated CK are more commonly reported in individuals with LD, especially in Dunnigan syndrome, the most frequent form of familial partial LD caused by variants in *LMNA*.^19^ However, the renal insufficiency and muscle cramps seen in our *POC5* cohort are more severe than what might be expected secondary to diabetes or LD.

Because we have evidence of diabetes mellitus with insulin resistance, variable LD features, and kidney disease, we recommend the following investigations in all individuals with biallelic *POC5* variants: yearly cardiovascular risk management screening, including blood pressure, lipids, glucose, insulin, liver and kidney function, ultrasound and fibroscan of the liver, urine albumin-to-creatinine ratio, and CK. When the diabetes mellitus, hepatic steatosis and/or hypertriglyceridemia are therapy resistant, we recommend considering a referral to a lipodystrophy expert center (https://www.eclip-web.org/) and treatment with recombinant human metraleptin.^18^

The available clinical data of participants varied because these were recruited from different medical specialists in various countries, and 2 participants were unavailable to follow-up. This may have resulted in an underestimation of the frequency of clinical features. Comprehensive clinical and genetic characterization of additional individuals with biallelic *POC5* variants is essential to delineate the full spectrum of manifestations associated with the *POC5*-related syndrome. Therefore, we established the website www.humandiseasegenes.nl/POC5 to collect detailed information from other individuals with pathogenic *POC5* variants.

Heterozygous variants in *POC5* have previously been linked to idiopathic scoliosis. Three recurrent variants (p.(Ala446Thr), p.(Ala455Pro), and p.(Ala429Val)) were identified in French families with adolescent idiopathic scoliosis (AIS).^7^ No additional phenotype description was provided on these individuals. In addition, a common variant (single-nucleotide variation [formerly polymorphism] rs6892146, chr5(GRCh38):g.75676165C>G NM_0010992 71.2:c.1585–1587G>C), located in intron 11 of *POC5,* was associated with scoliosis susceptibility in the Chinese population.^8^ This variant was reported to result in an increased *POC5* mRNA expression. In our cohort, scoliosis was observed in 2 of the participants with biallelic LoF *POC5* variants and treated with corset in childhood in 1. This suggests that also axial muscles can be involved in this disorder.

The pleiotropic effect of *POC5* variants on various organ systems can be explained by the ubiquitous expression of *POC5* (www.proteinatlas.org; last accessed August, 2024). A knockout mouse model of *Poc5* showed a significant impact on several organ systems, including the reproductive, endocrine, and neurological systems (www.mousephenotype.org, MGI: 1914713). Interestingly, the *Poc5* mouse knockout did not show an overt retinal phenotype contrary to humans and zebrafish.^1^ However, detailed phenotypic workup of the retina of this mouse model has not been performed, and retinal defects could emerge later in life or be present in a more subtle fashion because the mouse has a rod-dominated retina. POC5 plays an essential role in the elongation of the daughter centriole and is important for proper cell cycle progression. Previous studies showed that knockdown of POC5 by small interfering RNA in HeLa cells resulted in impaired cell cycle progression due to accumulation in the S phase. In addition, they showed that RPE cells treated with POC5 small interfering RNA lose POC5 localization in the daughter centriole.^5,20^ To investigate whether individuals with biallelic LoF variants in *POC5* show an aberrant localization pattern, we immunostained fibroblasts from P3, P9, and P10 for POC5. Indeed, we showed that the centriolar localization of POC5 was lacking or diminished in P3, P9, and P10 compared with controls (Figure 5A). Because of the nature of POC5 as a centrin-binding protein, we investigated the centriolar localization of centrin. Although the resolution of the imaging technique did not allow for a detailed analysis, a more diffuse centrin signal could be noted in fibroblasts from P3, P8, and P10, whereas control fibroblasts displayed distinct foci (Figure 5B). This finding suggests that LoF variants in POC5 may also effect the localization of centrin at the centrosome, although additional experiments are required to confirm this hypothesis. Given that POC5 is normally expressed at the basal body of primary cilia, we wondered whether cilium formation and/or length would be affected by the LoF variants in *POC5*. We did not observe a significant difference in ciliogenesis between participants and controls. Cilium length was normal for P9 and P10 but was shorter for P3. Because we only observed shorter cilia in cells from 1 of the participants, and the effect was modest, we are unsure about the significance of this finding in relation to *POC5-*associated disease. Interestingly, this patient also showed a more severe retinal phenotype and also had a homozygous variant of uncertain significance in *TULP1* (HGNC:12423, Chr6(GRCh38):g.35506268A>G NM_003322.6:c.828+6T>C). However, because TULP1 is barely expressed in fibroblast, a potential splice effect could not be investigated, but it is deemed unlikely that this *TULP1* variant causes the reduced cilium length in fibroblasts. Interestingly, cilia in osteoblasts from individuals with AIS carrying missense *POC5* variants were found to be shorter, in addition to the POC5 mislocalization that was observed.^9^ Investigation of the SHH pathway, one of the cilium signaling pathways, did not show differences in pathway activation between controls and participants (Figure 5E and F).

Ciliopathies are a clinically heterogeneous group of systemic disorders that can affect almost any organ of the human body.^6^ The retinal dystrophy that is observed in all participants with *POC5* variants aligns well in the known ciliopathy phenotypic spectrum. This also applies to the abnormalities of the kidney, although the cause of the renal insufficiency in the *POC5* families is not clear. Diabetes mellitus and insulin resistance are described in ciliopathies, such as Bardet-Biedl syndrome (BBS) and Alström syndrome, but are usually linked to obesity in these disorders.^21–23^ LD is thus far not a feature associated with ciliopathies. Both obesity and LD, however, have been linked to abnormalities in adipocyte function.^16^ Moreover, Alström syndrome has been described to have an intermediary phenotype between lipodystrophy and extreme obesity because of a low threshold of relative adipose failure,^24^ and in BBS, insulin resistance is out of proportion with the degree of obesity.^25^ Interestingly, adipocytes and obesity are connected through the primary cilium that is transiently present during adipogenesis. Previous studies have shown that confluent human and murine preadipocytes are ciliated, and that cilia are important for the induction of adipogenesis.^26,27^ Different signaling pathways required for adipogenesis take place in the primary cilium and its basal body, for instance, insulin-like growth factor (IGF-1), Wnt, and SHH.^26,27^ During adipogenesis, several BBS-associated genes showed a temporal and synchronized expression pattern implicating the importance of these genes during adipogenesis.^28^ Moreover, inhibition of 2 BBS-associated genes, *BBS10* (HGNC:26291) and *BBS12* (HGNC:26648), impaired ciliogenesis and activated proadipogenic pathways in fibroblast-derived adipocytes from individuals with BBS.^26^ Therefore, diabetes mellitus, insulin resistance, and LD may also be indicative of a ciliopathy.

Individuals with LoF *POC5* variants show significant similarity with Alström syndrome (retinal dystrophy, adipocyte dysfunction, insulin resistance, early-onset type 2 diabetes, nonalcoholic fatty liver disease, chronic progressive kidney disease, alopecia, polycystic ovarian syndrome, and scoliosis).^29^ Alström syndrome is caused by biallelic variants in *ALMS1* (HGNC:428).^30^ Similar to POC5, ALMS1 localizes to the centriole and the basal body of the cilium. A previous study showed that a 67% reduction of ALMS1 at the centrioles resulted in normal ciliogenesis and an increased cilium length.^31^ It is interesting to note that the ciliary phenotype upon knockdown of *ALMS1* in hTERT or *POC5* in proband-derived fibroblasts seems mild. We hypothesize that the function of these genes is highly tissue specific and therefore deem it interesting to study the cilium phenotype and function of POC5 in other cell types.

Alongside POC5, 2 other proteins, POC1A (HGNC:24488) and POC1B (HGNC:30836), localize to the distal centriole and likely also play a role in centriolar elongation.^20^ Biallelic variants in *POC1A* cause short stature, onychodysplasia, facial dysmorphisms, and hypotrichosis (SOFT) syndrome (OMIM 614813). The phenotypic spectrum has been expanded to also include insulin resistance, diabetes, and central fat distribution.^32^ This provides further evidence for the link between ciliopathies and adipose tissue disorders. SOFT syndrome is not clearly associated with retinal dystrophy, although a pigmentary retinopathy was recently observed in 3 individuals.^33,34^ Recessive variants in *POC1B* were previously linked to a nonsyndromic cone or cone-rod dystrophy.^35–38^ Today, extraocular abnormalities have been reported in individuals with *POC1B* variants, such as kidney disease and diabetes.^37,39,40^ Therefore, interdisciplinary collaboration is essential to define the full phenotype spectrum of individuals with variants in these genes.

In conclusion, we provide a detailed phenotype analysis of a cohort of 12 families with an autosomal recessive ciliopathy syndrome caused by LoF variants in *POC5*. This report expands the *POC5*-associated phenotype to include RCD, diabetes mellitus with severe insulin resistance, partial LD, renal failure, and muscle cramps. Furthermore, we provide evidence for the involvement of cilia in this syndrome. These findings highlight that adipose tissue abnormalities and metabolic dysfunction can be part of the ciliopathy phenotype spectrum.

## Supplementary Material

supplement

Additional Information

The online version of this article (https://doi.org/10.1016/j.gim.2025.101513) contains supplemental material, which is available to authorized users.

## Figures and Tables

**Table 1:** Clinical and genetic data of participants with biaLLeLic LoF variants in *POC5*

Family	1^a^	2	3	4	5	6	7	8	9	10	11	12	Total

Age (y)	19	59	26	46	32	47	57	35	27	22	54	38	
Sex	F	F	F	M	F	F	M	F	F	F	F	F	
Retinal dystrophy	+	+	+	+	+	−	+	+	+	+	+	+	11/12
Cataract	+	+	−	−	−	−	−	+	−	+	−	+	5/12
Diabetes mellitus	−	+	+	+	+	+	+	+	+	+	+	IR	10/12
Lipodystrophy	?	−	+	+	−	+	−	−	+	+	−	+	6/11
Hepatic steatosis	−	−	+	+	+	+	+	+	+	+	+	+	10/12
Puberty and gonadal disorders	+	+	+	+	+	+	−	+	+	+	−	−	9/12
Abnormality of the kidney	+	+	−	+	+	+	−	+	−	+	−	−	7/12
Abnormal muscle physiology	+	−	+	+	+	+	+	−	+	+	+	+	10/12
Abnormality of the skin and adnexa	+	−	+	+	+	+	−	+	+	+	−	+	9/12
POC5 variants^b^	p.(Asp102*) hmz	p.(Gln218*) hmz	p.[Asp229Glyfs*2, Asp229Gly] hmz	p.(Arg256*) hmz	p.(Arg256*) hmz	p.(Arg256*) hmz	p.(Trp296*) hmz	p.(Ala306Glufs*45) hmz	p.(Gln341*) hmz	p.(Lys352*) hmz	p.(Lys352*) hmz	p.(Ala354Serfs*12) pat p.(Glu198Asnfs*6) mat	

+, feature present; −, feature not noted/absent; *?*, feature uncertain; *F,* female; *hmz*, homozygous; *IR,* insulin resistance; *M*, male; *mat*, maternal; *pat*, paternal.

a PMID 29272404.

b NP_001092741.1.

## Data Availability

The pathogenic variant data are submitted to Leiden Open Variation Database. The genetic data are not publicly available because these could compromise research participant privacy. Genetic data may become available upon a data transfer agreement approved by local ethical committees. The RNA sequencing data were obtained in a diagnostic setting without consent to share these data with the scientific community. Participant sample identifiers from this study can be released upon request from “P1 to P11” to the corresponding local “DNA-number.” Specific variant requests or other data are available from the corresponding author upon request.
