# Supplementary material for: Bi-allelic loss-of-function variants in POC5 cause a syndromic retinal, endocrine, and neuromuscular ciliopathy
Source: Genet Med. Author manuscript; Available in PMC 2025 Nov 17. (PMC12622366; doi:10.1016/j.gim.2025.101513)
Supplement: supplement [file NIHMS2119735-supplement-supplement.docx]

**Supplement material to:**

**Bi-allelic loss-of-function variants in *POC5* cause a syndromic retinal, endocrine and neuromuscular ciliopathy**

Anneke T. Vulto-van Silfhout^1,2,^*, Ingrid M. Jazet^3^, Suzanne Yzer^4^, Jeroen Pas^4^, Serwet Demirdas^5^, Elisabeth F.C. van Rossum^6^, Alberta A.H.J. Thiadens^7^, Ronald van Beek^1^, Lonneke Haer-Wigman^1^, Daniela Q.C.M. Barge-Schaapveld^8^, Charlotte Brasch-Andersen^9^, Simon Frost^9^, Miriam Bauwens^10^, Elfride De Baere^10^, Irina Balikova^11^, Filip Van den Broeck^12^, Monika Weisz-Hubshman^13,14^, Pascal Joset^15^, Peter Miny^15^, Isabel Filges^15^, Susanne Kohl^16^, Pietro De Angeli^16^, Laura Kühlewein^17^, Jan-Philipp Bodenbender^17^, Tobias Haack^18^, Karin Poths^18^, Lidia Fernandez-Caballero^19,20^, Marta Corton^20^, Fiona Blanco Kelly^19,20^, Carmen Ayuso^19,20^, Peggy Martínez-Esteban^21^, John Vissing^22^, Jordi Díaz-Manera^23^, Volker Straub^23^, Ana Töpf^23^, Siying Lin^24,25^, Gavin Arno^24,25,26^, William L. Macken^27,28^, Jennifer Spillane^27^, Radha Ramachandran^29^, Erik de Vrieze^30^, Tjakko van Ham^5^, Susanne Roosing^1^, Machteld M. Oud^1^

^1^Department of Human Genetics, Radboud University Medical Centre, Nijmegen, the Netherlands.

^2^Department of Clinical Genetics, Maastricht University Medical Center, Maastricht, the Netherlands.

^3^Department of Medicine, Div. Endocrinology, Leiden University Medical Center, Leiden, The Netherlands.

^4^Department of Ophthalmology, Radboud University Medical Center, Nijmegen, the Netherlands.

^5^Department of Clinical Genetics, Erasmus University Medical Center, Rotterdam, the Netherlands.

^6^Department of Internal Medicine, Division of Endocrinology, Erasmus University Medical Center, Rotterdam, The Netherlands.

^7^Department of Ophthalmology, Erasmus University Medical Center, Rotterdam, The Netherlands.

^8^Department of Clinical Genetics, Leiden University Medical Center, 2333 ZA Leiden, The Netherlands.

^9^Department of Clinical Genetics, Odense University Hospital, Odense, Denmark.

^10^Center for Medical Genetics, Ghent University and Ghent University Hospital, Ghent, Belgium.

^11^Department of Ophthalmology, University Hospital Leuven, Leuven, Belgium.

^12^Department of Ophthalmology, Ghent University Hospital, Ghent, Belgium.

^13^Department of Molecular and Human Genetics, Baylor College of Medicine, Houston, Texas, USA

^14^Texas Children's Hospital, Houston, Texas, USA

^15^Medical Genetics, Institute of Medical Genetics and Pathology, University Hospital Basel, Basel, Switzerland.

^16^Molecular Genetics Laboratory, Institute for Ophthalmic Research, Center for Ophthalmology, University, Tübingen, Germany

^17^University Eye Hospital, Center for Ophthalmology, Eberhard Karls University, Tübingen, Germany

^18^Institute for Medical Genetics and Applied Genomics, Eberhard Karls University, Tübingen, Germany

^19^Department of Genetics & Genomics, Instituto de Investigación Sanitaria-Fundación Jiménez Díaz University Hospital, Universidad Autónoma de Madrid (IIS-FJD, UAM), Madrid, Spain

^20^Center for Biomedical Network Research on Rare Diseases (CIBERER), Instituto de Salud Carlos III, Madrid, Spain

^21^Neurofisiologia Clínica, Instituto Nacional de Salud del Niño San Borja, Lima, Perú.

^22^Copenhagen Neuromuscular Center, Rigshospitalet, University of Copenhagen, Copenhagen, Denmark

^23^The John Walton Muscular Dystrophy Research Centre, Translational and Clinical Research Institute, Newcastle University and Newcastle Hospitals NHS Foundation Trust, Newcastle upon Tyne, UK

^24^ NIHR Biomedical Research Centre, Moorfields Eye Hospital and the UCL Institute of Ophthalmology, London, UK

^25^ UCL Institute of Ophthalmology, University College London, London, UK

^26^ Division of Research, Greenwood Genetic Center, Greenwood, South Carolina, USA

^27^ Department of Neuromuscular Diseases, UCL Queen Square Institute of Neurology, London WC1N 3BG, UK

^28^ NHS Highly Specialised Service for Rare Mitochondrial Disorders, Queen Square Centre for Neuromuscular Diseases, The National Hospital for Neurology and Neurosurgery, London WC1N 3BG, UK

^29^ Department of Adult Inherited Metabolic Diseases, Metabolic Medicine and Chemical Pathology, Guys and St Thomas' Hospitals NHS Foundation Trust, London, UK

^30^ Department of Otorhinolaryngology, Radboud University Medical Center, Nijmegen, Gelderland, Netherlands.

**Table of contents**

1. Targeted and transcriptome-wide RNA results
   - Supplemental Table 1
   - Supplemental Figure 1
   - Supplemental Table 2
2. Detailed genetic and clinical data of participants with bi-allelic LoF variants in POC5
   - Supplemental Table 3
3. Supplemental descriptions
   - Participant 1
   - Participant 2
   - Participant 3
   - Participant 4
   - Participant 5
   - Participant 6
   - Participant 7
   - Participant 8
   - Participant 9
   - Participant 10
   - Participant 11
   - Participant 12
4. Results immunofluorescence
   - Supplemental Figure 2
   - Supplemental Table 4
5. Materials and methods
   - Targeted and transcriptome-wide RNA analysis
   - Immunofluorescence
6. References
7. **Targeted and transcriptome-wide RNA results**

**Supplemental Table 1.**

*POC5* RT-PCR primer sequences:

| **Name** | **Position** | **Sequence** |
| --- | --- | --- |
| *POC5* forward primer | Exon 2 | CTCCAGTCGAGGCAGTTCTG |
| *POC5* reverse primer | Exon 12 | AGGATGAATGGTCCGGGGAT |

Expected product size: 1587bp


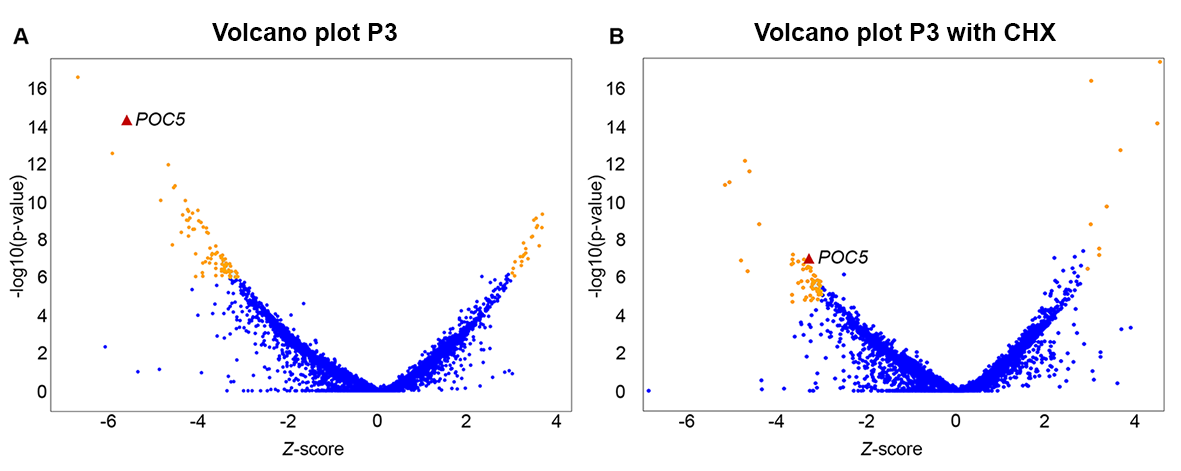


**Supplemental Figure 1**.

Expression outlier genes for P3 compared to control samples. Homozygous missense variant (c.686A>G) in *POC5* leads to 4-bp deletion (r.686_689del) in the POC5 transcript that in turn results in a reduced expression and nonsense-mediated decay of POC5. A) Volcano plot of P3 showing expression outlier genes in orange with Z-score >3 and p-value <0.01. POC5 has a p-value of 5.9e-07 and a Z-score of -5.60. B) Volcano plot of P3 with cycloheximide (CHX) treatment showing expression outlier genes in orange with Z-score >3 and p-value <0.01. POC5 has a p-value of 1.1e-03 and a Z-score of -3.27.

**Supplemental Table 2.**

*POC5* qRT-PCR primer sequences:

| **Name** | **Position** | **Sequence** |
| --- | --- | --- |
| *POC5* forward primer 2 | Exon 10 | TCCTGTTTCTGCTCTTGGTGC |
| *POC5* reverse primer 2 | Exon 11 | TCCGGGCTGTAATAGTTCTTCC |

1. **Detailed genetic and clinical data of participants with bi-allelic LoF variants in *POC5***

**Supplemental Table 3.** **Detailed genetic and clinical data of participants with bi-allelic LoF variants in *POC5***

| *Family* | 1^c^ | 2 | 3 | 4 | 5 | 6 | 7 | 8 | 9 | 10 | 11 | 12 |
| --- | --- | --- | --- | --- | --- | --- | --- | --- | --- | --- | --- | --- |
| Age (years) | 19 | 59 | 26 | 46 | 32 | 47 | 57 | 35 | 27 | 22 | 54 | 38 |
| Sex | Female | Female | Female | Male | Female | Female | Male | Female | Female | Female | Female | Female |
| Origin | Moroccan/Yemenite Jewish | Swiss | Suriname | Peru | Turkish cypriot | Turkish cypriot | Iraq | Iraq | Afghanistan | Turkey | Russia | Spain |
| Genomic variants (GRCh 38) | g.75705707_ 75705708del | g.75694693G>A | g.75694659T>C | g.75692425G>A | g.75692425G>A | g.75692425G>A | g.75690471C>T | g.75690442_ 75690445del | g.75689120G>A | g.75689087T>A | g.75689087T>A | g.75689087dup g.75694755_ 75694758del |
| cDNA variants^a^ | c.304_305del | c.652C>T | c.686A>G | c.766C>T | c.766C>T | c.766C>T | c.887G>A | c.917_920del | c.1021C>T | c.1054A>T | c.1054A>T | c.1059dup, c.592_595del |
| GnomAD (v4.1; het alleles) | absent | absent | absent | 9/1592440 | 9/1592440 | 9/1592440 | 2/1611268 | 2/1612538 | 2/1580900 | absent | absent | 1/1602998, 1/1597014 |
| Protein change^b^ | p.(Asp102*) hmz | p.(Gln218*) hmz | p.[Asp229Glyfs*2,Asp229Gly] hmz | p.(Arg256*) hmz | p.(Arg256*) hmz | p.(Arg256*) hmz | p.(Trp296*) hmz | p.(Ala306Glufs*45) hmz | p.(Gln341*) hmz | p.(Lys352*) hmz | p.(Lys352*) hmz | p.(Ala354Serfs*12) paternal  p.(Glu198Asnfs*6) maternal |
| Other variants | - | - | *TULP1* (VUS) c.828+6T>C hmz | - | - | - | - | - | - | *ARMH1* (VUS) p.(Asp73fs) *dn* | - |  |
| *Growth* |  |  |  |  |  |  |  |  |  |  |  |  |
| Height (m (SD)) | 1.43 (-3.4) | 1.59 (-0.8) | 1.61 (-0.4) | 1.56 (-3.4) | 1.58 (-1) | 1.56 (-1.2) | 1.65 (-1.6) | 1.50 (-2.3) | 1.60 (-0.6) | 1.64 (0) | 1.56 (-1.3) | 1.63 (+0.1) |
| Weight (kg (SD for height)) | NA | 63 (+1.5) | 52 (-1.1) | 39 (-1.5) | 65 (+1.7) | 57.3 (+0.9) | 68 (+1.7) | 51 (+0.3) | 41 (-2.2) | 47 (-1.5) | 66 (+1.9) | 57 (+0.3) |
| BMI (kg/m^2^) SD)) | NA | 25 | 20 | 16 | 26 | 24 | 25 | 23 | 16 | 18 | 27 | 22 |
| HC (cm (SD)) | (-3.8) | NA | NA | NA | NA | NA | 55 (-1.6) | NA | NA | 52.7 (-1.6) | NA | NA |
| *Eye* |  |  |  |  |  |  |  |  |  |  |  |  |
| Retinal dystrophy | RP | RP | RCD | RP | RCD | - | RP | RP with MD | RP | RP | RP | RP |
| Age of onset | 14y | School age | 2-3y | 2nd decade | 26y | NA | 4th decade | 10-12y | 3rd decade | 10y | Childhood | 16-17y |
| BCVA | 0.5 | Legally blind | LP | Blind | 0.3 | NA | LP | 0.8/0.5 | 1.0 | 1.0 | 0.2 | 1.0 |
| Abnormality of refraction | Myopia at age 6y | - | - | - | RE S +0.00 x C -3.50 x 100, LE S +1.00 x C -4.75 x 080 | Reading glasses | - | RE S -1.00, LE S +1.25 x C -0.50 x 031 | RE S -4.00, LE S -4.00 | RE S -4.50, LE S -6.50 | RE S +2.75, LE S +3.50 | RE S -0.50 x C  -0.50 x 078, LE S -0.50 x C -1.00 x 068 |
| Cataract | Mild central posterior subcapsular | Bilateral at age 40y | - | - | - | - | - | + | - | + | - | RE |
| Other | - | - | Nystagmus at age 3y | - | Keratoconus | - | - | - | - | - | - | - |
| *Endocrinologic* |  |  |  |  |  |  |  |  |  |  |  |  |
| Diabetes mellitus (age of onset) | U | Type 2 (40y) | Type 2 (25y) | Type 1 (20y) | Type 2 (13y) | Type 2 (early 20s) | Type 2 (49y) | Type 2 (26y) | Type 2 (20y) | Type 2 (15y) | Type 2 (46y) | Insulin resistance |
| Lipodystrophy | Very thin habitus | Not noted | Thin limbs, abdominal fat deposit | Increased muscle mass | Not Noted | Loss of subcutaneous fat arms, symmetric fat distribution thighs | Not noted | Not noted | Thin limbs, abdominal fat deposit | Loss of subcutaneous fat face and limbs | Not noted | Fat on upper segment, loss of fat from the extremities |
| Abnormality of the liver | U | U | Hepatic steatosis | Hepatic steatosis | Hepatic steatosis | Hepatic steatosis | Elevated transaminases | Hepatic steatosis | Hepatic steatosis | Hepatic steatosis | Hepatic steatosis | Hepatic steatosis |
| Puberty and gonadal disorders | Early fast puberty | Ovary cyst | PCOS | Low testosterone level | Oligomenorrhea | Irregular menstruations, PCOS | - | Ovary cysts | Irregular menstruations, PCOS | Irregular menstruations, oligomenorrhea | - | - |
| *Other* |  |  |  |  |  |  |  |  |  |  |  |  |
| Abnormality of the kidney | Recurrent glomerulonephritis | Renal insufficiency, kidney Tx 37y | - | Renal failure | Single functional kidney since birth | Renal insufficiency | - | Renal insufficiency | - | Albuminuria | - | - |
| Neuromuscular abnormalities | Painful muscle cramps, elevated CK | - | Proximal muscle weakness | Muscle cramps since age 7y, fasciculations | Muscle cramps since age 13y, elevated CK, suspected of rhabdomyolysis | Muscle cramps since age 26y, increasingly severe, elevated CK, finger weakness | Muscle cramps, elevated CK, muscle biopsy myopathic changes | - | Dyskinesia, muscle cramps | Muscle cramps since age 15y | Muscle cramps, neuropathy | Fasciculations and cramps since infancy, abnormal EMG,  paraesthesia |
| Skeletal abnormalities | - | Unspecified joint and orthopedic complaints | Scoliosis | Large hands and feet, brachydactyly | Large hands | Large hands, mild kyphosis | Brachydactyly | - | - | Mild pectus excavatum, hip dysplasia, large hands and feet | Osteoarthrosis hands/feet, degenerative back pain | Scoliosis |
| Heart abnormalities | - | - | Small Q in lead III and slow R progression | - | Borderline concentric left ventricular hypertrophy | Mild concentric hypertrophy | - | - | - | - | - | - |
| Facial dysmorphism | Wide mouth, prognathia, midface hypoplasia | - | High palate | Wide nasal base, wide mouth, prognathia, large ears | - | - | - | - | - | Prominent nose, wide mouth, prognathia, large ears | - | Micrognathia |
| Hair abnormalities | - | - | Beginning alopecia totalis, sparse eyebrows and eyelashes | Sparse hair, thin eyebrows, no eyelashes | Thin scalp hair, hirsutism on the face | Thin and brittle scalp hair | - | Hair loss, thin eyebrows, absent eyelashes | Hirsutism lower back | Hirsutism, thick eyebrows, sparse and thin scalp hair | - | Thin and sparse hair, thick eyebrows |
| Skin abnormalities | Soft skin | - | Acanthosis nigricans, striae | Thick skin | Easy scarring, pigmented | Dry skin | - | Acanthosis nigricans | Acanthosis nigricans | Acanthosis nigricans, soft skin with atypical scarring | - | - |
| Other | - | GI abnormalities, macrocytic anemia, depression | Anorexia | Conical teeth, ectodermal dysplasia | Death due to acute pancreatitis and multiorgan failure at 32y with highly elevated triglycerides | Brittle nails, hypothyroidism, highly elevated triglycerides, hypertension, chronic diarrhea, dyslexia | - | Misalignment of deciduous teeth | Conductive hearing loss, congenital fusing of auditory ossicles | Swallowing problems, splenomegaly, lactose intolerance, deep voice | - | Delayed eruption and misalignment of secondary teeth, antithyroid antibodies |
| *Family history* |  |  |  |  |  |  |  |  |  |  |  |  |
| Consanguinity | no | small community | no | yes | small community | small community | yes | yes | yes | yes | no | no |
| Affected family members | - | Two brothers with renal failure and vision impairment | - | - | One brother muscle cramps | Diabetes in brother, mother and maternal grandfather, cramps in paternal aunt | Three siblings with muscle cramps, RP and diabetes mellitus, two died of renal failure | Brother with visual impairment | Brother with RP, diabetes and muscle cramps, two sisters with blindness and diabetes | - | Renal failure in sibling, diabetes mellitus in mother and two siblings | - |

BCVA: best corrected visual acuity; CK: creatine kinase; CRD: cone-rod dystroph; *dn*: *de novo*; HC: head circumference; het: heterozygous, hmz: homozygous; LCA: Leber congenital amaurosis; LE: left eye; LP: light perception; MD: macular degeneration; N: normal; NA: not available; NR: not reported; PCOS: polycystic ovary syndrome; RCD: rod-cone dystrophy; RE: right eye; RP: retinitis pigmentosa; Tx: transplantation; U: unknown; VUS: variant of uncertain significance; y: years, +: present; -: absent. ^a^: NM_001099271.2, ^b^: NP_001092741.1, ^c^: PMID 29272404

1. **Supplemental descriptions**

**Participant 1 – *POC5*** g.75705707_75705708del c.304_305del p.(Asp102*) homozygous

This individual was published previously.^1^ The proband was a 19-year-old girl, born to healthy non-consanguineous Moroccan/Yemenite Jewish parents. She has one unaffected brother. Prenatal history is non-contributary. Birth weight was 2400 grams (<p3). In childhood she was diagnosed with early fast puberty with normal hormonal profile. In addition, she started to have recurrent glomerulonephritis episodes that manifested by hematuria and proteinuria without elevation in blood pressure. Kidney biopsies showed first time focal segmental glomerulonephritis and IGA nephropathy at the second time. She was treated with ACE inhibitors. Kidney ultrasound was normal. She also had recurrent episodes of elevated creatine phosphokinase (CPK) and muscle pain/cramps. Electromyogram and CPK levels were normal between episodes. Metabolic evaluation, echocardiogram and electrocardiogram were normal. Myopia was diagnosed at age 6 years, and later mild central posterior subcapsular cataract / polar cataract was identified. At age 14 she was diagnosed with retinitis pigmentosa (RP). Full-field electroretinography (ffERG) revealed non-detectable rod responses, but there was still a sizable photopic cone response: 30 Hz cone flicker amplitudes of around 20 microvolt amplitudes (our lower limit of normal being 60 microvolts) with a delayed implicit time of 38-40 msec (upper limit of normal being 33 msec). Visual acuity at age 19 was 20/40. Goldmann visual fields constricted to 10-20 degrees from fixation with the IV4e target. Fundus findings included salt and pepper-like hyperpigmentation in the midperiphery accompanied by early bone-spicule-like pigmentary changes, mild optic disc pallor, and mild attenuation of retinal vessels.^1^

Physical examination at 19 years of age showed thin habitus, short stature (-3.4 SD) and microcephaly (-3.8 SD). She had midface hypoplasia, wide mouth, and prominent chin, but no other significant dysmorphic features.

**Participant 2 – *POC5*** g.75694693G>A c.652C>T p.(Gln218*) homozygous

The proband was seen at the age of 59 years. A loss of vision was already obvious at school age. She successfully attended a regular school and later worked in an office as a telephone operator without formal professional training. Today she is legally blind due to progressing RP.

At the age of 29 a declining renal function was first noticed and at age 37 she received a kidney donated by a healthy older sister. Diabetes mellitus was diagnosed at 40 years and treated with oral antihyperglycemic agents for some years before she became insulin dependent. At the age of 58 the proband suffered from a transient ischemic attack (TIA) affecting the downstream area of the arteria cerebri posterior. Ever since she has been treated for hypertonia and mild dyslipidemia. The medical history also includes interventions such as bilateral cataract surgery in her late forties, cholecystectomy and removal of an ovarian cyst in her mid-forties, gastrointestinal conditions such as an antral gastritis, sigmoid diverticulosis, and polypoid changes of the colon mucosa as well as currently unspecified joint and other orthopedic complaints, a macrocytic normochromic anemia and depressive episodes.

Her actual height is 1.59 m (-0.8 SD; mother 1.59 m; father 1.82 m), weight 63 kg (BMI 25 kg/m^2^). There were no obvious physical anomalies.

The proband is the youngest in a sibship of 12 children. Her father was 59 and healthy when he lost his life due to an accident, her mother died at 85 without suspicious symptoms. The parents were not known to be consanguineous, but originated for a small community. One brother died in his mid-thirties due to renal failure, another one at 42 years after an unsuccessful renal transplantation. Both also suffered from a vision impairment. A further brother died at 46 due to an accident and another one at 10 weeks for unknown reasons. The proband has a 36-year-old healthy daughter. The affected brothers did not have children.

Exome sequencing was performed using the Twist Comprehensive Exome Kit (Twist Bioscience, South San Francisco, CA, USA), followed by paired-end sequencing of 150 bp forward and 150 bp reverse using a NextSeq 500 System (Illumina, San Diego, CA, USA). Raw fastQ files were aligned to the hg19 reference genome using the Varsome Clinical software (Saphetor SA, Lausanne, Switzerland). Average sequencing coverage was 120-fold. Variants (incl. exon/intron-boundary, +/-10 bps) were called if observed in at least 20% of reads with sufficient quality level and with an overall minor allele frequency below 2% (gnomAD). Variants of known indication relevant genes for Bardet-Biedl syndrome, renal diseases, ciliopathies and eye disorders (GeneReviews, Genomics England (green/amber)-Panels) were further manually assessed for possible association with a phenotypic overlap with the proband (a. o. Clinvar, gnomAD, HGMD Professional (Human Genome Mutation Database, http://www.hgmd.org/), Alamut Visual Plus v.1.2 (Sophia Genetics), OMIM, PubMed). The homozygous POC5 variant was confirmed by Sanger sequencing using an ABI capillary sequencer (Thermo Fisher Scientific, Massachusetts, USA).

**Participant 3 – *POC5*** g.75694659T>C c.686A>G p.[Asp229Glyfs*2,Asp229Gly] homozygous

The proband is a 22-year-old female of Surinamese descent. Her parents were non-consanguineous. Her mother died at age 30 due to liver cancer (no detailed information available).

She was born after a pregnancy marked by repeated illnesses of her mother (specifics unknown) at approximately 40 weeks of gestation with an estimated normal birth weight. Developmentally, her first year was unremarkable; she began walking at around one year and displayed normal speech development.

Between ages 2-3 years, her parents noted roving eye movements and poor vision. At the age of 9 years, her visual acuity was counting fingers from 0.5-1 meter. At that time, she had an electroretinogram which showed absent scotopic and 10% photopic responses. Goldmann visual fields showed a central 10 degrees. On OCT a thickened interna limitans membrane was noted, right eye (RE) more than left eye (LE). She was subsequently diagnosed with an early-onset rod-cone dystrophy or Leber congenital amaurosis. At ophthalmologic examination at the age of 22 years, she presented with light perception in both eyes. Slit lamp examination revealed a congenital nystagmus (both horizontal and vertical), small lenticular opacities and corneal verticillata. Fundoscopy revealed well-demarcated normal colored optic discs, normal maculae, attenuated vessels and bone spicules with fine white intraretinal flecks in all quadrants. Inferiorly in the RE, there is also an atrophic spot.

Since the age of 2, she exhibited a very slim build, and at age 10, she underwent analysis for her low body weight despite normal or slightly increased appetite, with no signs of malabsorption, infection, or other chronic disease. At age 22, she was referred to the internist due to swelling and pain in her hands, arms, face, abdomen, and legs, accompanied by proximal muscle weakness. Despite being consistently slim, she recently gained 5 kg in weight, especially in her abdominal region. Additionally, she reported secondary amenorrhea since discontinuing oral contraceptives (having experienced normal menstrual cycles between ages 12-20), along with occasional acne.

Physical examination showed a height of 1.61 m (-0.4 SD), weight of 52 kg (-1.1 SD), BMI of 19.9 kg/m2, with subtle central adiposity. She had beginning alopecia totalis, sparse eyebrows, a high palate, an enlarged liver, and scoliosis. A prominent axillary and inguinal acanthosis nigricans, as well as acanthosis in the neck was observed, as well as extensive striae on the thighs, the axilla and the breasts.

Laboratory tests revealed hyperinsulinism (1076 pmol/L (ref range 12-96 pmol/L)) with normal fasting glucose and leptin levels, elevated liver enzymes, and an abnormal lipid profile. She had an elevated testosterone and transvaginal ultrasound showed polycystic ovaries leading to a diagnosis of polycystic ovary syndrome. Abdominal ultrasound showed severe liver steatosis and prominent subcutaneous adipose tissue distribution (ventral of the abdominal muscles). There was no microalbuminuria and tension was normal. Renal and cardiac ultrasound revealed no abnormalities. ECG showed a small Q in lead III and slow R progression.

She initiated metformin, titrated to twice daily 1000 mg, alongside lifestyle guidance focusing on a healthy diet and exercise regimen. She exhibited a very good response, with notable decreases in insulin and testosterone levels, reversal of liver steatosis, and resolution of her complaints of swollen limbs.

Genetic testing of *LMNA* (HGNC:6636) revealed no abnormalities. Exome sequencing was performed on the Illumina HiSeq after enrichment with the Agilent SureSelectXT Human All Exon 50Mb Kit. Read alignment was performed using BWA and variant calling using GATK (SNVs) and CoNIFER (CNVs). Initial clinical exome analysis identified a homozygous variant of unknown significance in *TULP1* (HGNC:12423, c.828+6T>C, p.?). Clinical transcriptome analysis in RNA from fibroblasts showed decreased expression of *POC5*, and a 4 bp deletion in remaining *POC5* transcript. This assisted in prioritizing the homozygous variant in *POC5* (c.686A>G p.[Asp229Gly;Asp229Glyfs*2]), as a cause of skipping part of exon 6 of *POC5*, leading to a frameshift.^2^ The effect of the *TULP1* variant could not be further investigated due to the low expression levels.

**Participant 4 – *POC5*** g.75692425G>A c.766C>T p.(Arg256*) homozygous

The proband is a 46-year old male. He is the son of consanguineous parents with an unremarkable family history. He was born preterm at approximately 32 weeks of gestation and had normal psychomotor development. Symptoms started at the age of 7 years with non-painful muscular contractions at first in both legs, that later became painful and debilitating. These contractions were triggered by physical effort and worsened in cold season; they also improved in warm weather. Patient also noticed muscle rippling. He was diagnosed with cramps and fasciculations. He has preserved muscle balance and sensitivity and normal deep tendon reflexes. Also, motor and sensory conduction studies were normal. Fasciculation, cramps and myokymic discharges with normal motor unit action potentials were seen on electromyography. CK levels were normal. At the age of 26 years, he began treatment with phenytoin for his cramps, but was interrupted after a couple of years as no significant improvement was noted.

Since the age of 12 years, he had a decrease in vision, predominantly at night. He was diagnosed with RP at the age of 19 years. Visual acuity progressively worsened and he is now legally blind.

The proband was evaluated by a dermatologist due to his thick skin with scarce hair. In addition, conical teeth were observed and ectodermal dysplasia was diagnosed.

At the age of 25 years he was diagnosed with diabetes mellitus type 1 with insulin resistance and is currently treated with insulin. Because of kidney failure he is treated with enalapril. An abdominal ultrasound showed hepatic steatosis, and he had low testosterone level. Echocardiogram was normal.

The physical appearance at that age was a generalized increase in muscle mass, as if he was exercising/weightlifting (which was not the case). Over the years he showed considerable decrease of muscle mass and weight with a lipodystrophic appearance and acromegaly. He now as a height of 1.56 m (-3.4 SD) and weight of 39 kg (-1.5 SD, BMI 16 kg/m^2^). He had a wide nasal base, scarce hair on the scalp, large ears, mandibular prognathia, conical teeth and thick skin.

The participant was included in the Latin-SEQ project (<https://latin-seq.com/>). Exome sequencing was carried out at the Centro Nacional de Análisis Genéticos (CNAG, Spain) and the processed data uploaded to the Genome-Phenome Analysis Platform (GPAP, <https://platform.rd-connect.eu>). Initially, a panel of 672 genes associated with neuromuscular conditions failed to identify any putative causative variants. Further genome-wide re-analysis searching for homozygous high impact, extremely rare variants revealed the nonsense variant in *POC5*.

**Participant 5 – *POC5*** g.75692425G>A c.766C>T p.(Arg256*) homozygous

The proband (GC28159) is a 32-year-old female. She was born at full term and had a normal early motor and cognitive development. She first developed muscle pain around the age of 13. This presented with muscle cramps in her arms and legs the day after activity (dancing) and the pain persisted the following day. Since then she has been developing recurrent episodes of cramps, of variable length, lasting up to about 48 hours. Cramping occurred weekly until the patient started taking Quinine age 30 years. She currently experiences cramps once a month and when she gets them they last around 10 minutes. She has had an episode of suspected rhabdomyolysis with severe pain and CK >10,000. Cramps are triggered after exercise and cold weather, but are not associated with fever, infection or fasting. There is no association of cramps with fever or infection or fasting. She does not have episodes of dark urine. MRI of her lower limbs did not demonstrate signs of a myopathy. A muscle biopsy showed mild fibre size variation due to preferential fast fibre atrophy, single necrotic fibre and widespread unevenness of oxidative staining in both fibre types and occasional well defined core lesion and overall preserved fibre typing, consistent with the clinical presentation of rhabdomyolysis and limited physical activity.

Menarche occurred at age 15 years. Her periods were irregular and stopped at 18-19 years. She now has oligomenorrhoea. She has been diagnosed with polycystic ovarian syndrome. She has hirsutism and sparse scalp hair. She was diagnosed with fatty liver and type two diabetes in adulthood.

The patient was referred for cardiac surveillance due to a suspected diagnosis of mitochondrial disease or LCHAD deficiency, both of which are associated with an elevated risk of cardiomyopathy. The patient also presented with borderline blood pressure and reported experiencing palpitations with light-headedness but no blackouts. Initial investigations revealed mild left ventricular hypertrophy (LVH) on ECG. A cardiac MRI conducted showed borderline concentric LVH with a maximum wall thickness of 11-12 mm in the basal and mid-septum, normal cardiac function, and no evidence of fibrosis or scarring. An echocardiogram indicated a normal left ventricular size and function, with a left ventricular ejection fraction of 55%, wall thickness at the upper limit of normal, and no valvular pathology. A Holter monitor study did not show any arrhythmias.

She presented to the ophthalmic genetics service at age 28 due to progressive worsening of night vision starting 2 years prior, and loss of visual field starting 10 months prior. She reported on significant difficulty adjusting to varying light levels, requiring more time to adapt. Ophthalmic examination showed visual acuities of 6/18 Snellen in the RE, and 6/36 Snellen in the LE, correcting to 6/18 with pinhole. She had astigmatism, with refraction of S +1.00 x C-4.75 x 080 in the RE and S +0.00 x C-3.50 x 100 in the LE. Ishihara plate testing revealed no significant colour vision deficiency. Slit lamp anterior segment examination showed Vogt’s striae and a cone corneal contour in both eyes, consistent with keratoconus. Both eyes had clear visual axis with no significant cataract. Fundus examination showed pigmentary mottling and mild pigment spicules in the mid-peripheral retina, along with retinal vascular attenuation. Ultra-widefield autofluorescence imaging (Optos) displayed speckled hypoautofluorescence in the mid-peripheral retina and a hyperautofluorescent “Robson” ring at the posterior pole. OCT scans showed a mild epiretinal membrane in both eyes, as well as loss of ellipsoid zone in the far perifoveal region with a normal foveal structure. Electroretinography was consistent with severe generalized retinal dysfunction affecting rod slightly more than cone photoreceptors, in keeping with a severe rod cone dystrophy, with severe macular involvement bilaterally. On neurological examination there is mild drooping of right eyelid but no frank ptosis. She does not have ophthalmoplegia, or facial weakness. Limb power, tone, reflexes, coordination, and sensation are normal, apart from mildly reduces Achilles tendon reflexes. Romberg's sign is negative. Her height was 1.58 m (-1 SD) and her weight 65 kg (+1.7 SD). She died suddenly at the age of 32 years due to acute pancreatitis and multiorgan failure. Triglycerides were highly elevated.

Her parents are not knowingly related, but originated from a small Turkish Cypriot community, and are. They are in their 50s and 60s and do not have any night vision problems or visual field loss. She has an older and a younger brother, neither of whom have vision problems. Her younger brother occasionally has mild myalgia after exercise. He is very active and has never had severe symptoms. He has not presented for testing. There is no known history of similar visual problems in her extended family.

Previous genetic testing included microarray-based comparative genomic hybridization, targeted gene panel testing for retinal dystrophy, glycogen storage disease, and rhabdomyolysis, and whole mitochondrial sequencing in blood, which did not identify a definitive molecular diagnosis. A single heterozygous variant of uncertain significance in the *HADHA* gene (HGNC:4801, NM_000182.5) c.316A>G p.(Met106Val) was noted. Biallelic variants in *HADHA* are associated with long-chain 3-hydroxyacyl-coA dehydrogenase (LCHAD) deficiency, which shares some clinical features with those noted in the patient, including liver dysfunction, cardiomyopathy and retinopathy.^3^ Although a second pathogenic variant in *HADHA* was not identified, a skin biopsy was performed to investigate for LCHAD and long chain thiolase deficiency but showed no abnormalities.

Genome sequencing was subsequently performed with virtual gene panel analysis using the PanelApp “R98 Likely inborn error of metabolism - targeted testing not possible” panel v4.0, as well as the “R381 Other rare neuromuscular disorders” panel v23.7, inclusive of 731 and 214 genes respectively.^4^ This did not identify any relevant findings of note. The patient has a single likely pathogenic variant in *OBSCN* (HGNC:15719, NM_001386125.1) c.21934+1G>A*,* however, extensive screening of the gene has not identified a second likely disease-associated variant, and the patient is considered to be heterozygous for a pathogenic variant for this condition (Susceptibility to Rhabdomyolysis 1, autosomal recessive, OMIM # 620235).

A further re-analysis of the genome sequencing data identified homozygosity for a stopgain variant in *POC5*, (NM_001099271.2) c.766C>T p.(Arg256*).

**Participant 6 – *POC5*** g.75692425G>A c.766C>T p.(Arg256*) homozygous

The patient is a 48 year old female who presented with acquired myotonia with severe cramps and muscle rippling. In her mid-twenties she began experiencing muscle cramps in her lower limbs which were precipitated by exercise. For example, she would have to lie down after dancing due to the severity of her cramps. Her symptoms gradually worsened in severity, and she was admitted to the hospital on several occasions in her 30s with severe cramps and raised CK. Her cramps became more widespread (involving her upper limbs) and were associated with prominent fasciculations and muscle rippling. On examination, the patient exhibited fasciculations and muscle spasms with rippling and sustained indentations with some muscle fibres visibly contracted and others less so. Most EMGs were consistent with nerve hyperexcitability. There was evidence of myokymia with brief frank neuromyotonic discharges. Rapidly firing neurogenic units have been found during cramps. Nerve conduction studies showed carpal tunnel syndrome. Nerve excitability studies were consistent with depolarisation. Extensive antibody testing was negative (LG1, CASPR2, GAD, Gly, anti-neuronal, VGCC, AchR, dsDNA, lupus anticoagulant, ANCA, C3,C4, ESR) or only weakly positive (ANA, ENA, GD1b, Gq1b) on multiple occasions, which did not give a clear cause for her symptoms. The patient has had two muscle biopsies. The first showed subtle and non-specific variably sized vacuoles in scattered fibres but not definitely pathological. A second muscle biopsy demonstrated Type 2 fibre atrophy with no evidence of an inflammatory myopathy. Creatine kinase varies between 400s and one peak at 1200. An cerebral MRI in 2018 was normal. Trials of pregabalin, baclofen, diazepam, exiletine and quinine have not been effective. She has been treated with to IVIg, plasma exchange, and Rituximab over the last ten years with some response. In between attacks she is relatively well but relapses are severe.

She was well as a child with normal development and had no issues with part taking in sports or exercise. She did not have learning difficulties apart from dyslexia. The patient developed PCOS in her late teens and had irregular periods until her late thirties when she developed amenorrhea. In her early twenties she was diagnosed with type two diabetes, which has been difficult to control. She currently takes metformin, linagliptin and was started on long acting insulin in her 40s. She has an unusual fat distribution which is very asymmetric in her thighs and she has reduced subcutaneous fat in her arms. The patient has large hands which continued to grow in adulthood. She has chronic renal impairment and hypertension. She takes three antihypertensive medications. She was diagnosed with hypothyroidism in her 30s. She has hypercholesterolemia and elevated triglycerides for which she is treated with fibrate. Cardiac examination revealed a mild concentric hypertrophy. She has also been diagnosed with ANA positive polyarthralgia. She has chronic diarrhoea. There were no visual problems, except for reading glasses. She had a height of 1.56 m (-1.2 SD) and a weight of 57.3 kg (0.9 SD). There were no obvious facial dysmorphisms. She had a kyphosis. The patient has always had thin scalp hair. Her hair has become increasingly sparse and brittle in older age and she also has brittle nails and a slightly dry skin.

The patient is of Turkish Cypriot origin, her parents are not knowingly related but are from the same village. She has a strong family history for type two diabetes in her brother, mother and maternal grandfather. Her paternal aunt and some paternal second cousins have a history of less severe muscle cramps. They were not available for testing.

Genome sequencing as a singleton was undertaken (via the UK 100,000 Genomes Project) with gene panels for brain channelopathy, hereditary ataxia, skeletal muscle channelopathy and mitochondrial disorders (panel content details available at <https://panelapp.genomicsengland.co.uk/panels/>) and the *POC5* variant was detected

**Participant 7 – *POC5*** g.75690471C>T c.887G>A p.(Trp296*) homozygous

The proband is a 57-year-old male. He has two healthy children. He comes from a family of eight siblings, of which four (including himself) are affected, all with severe muscle cramps with elevated CK, RP (two of them completely blind) and diabetes. Two of the affected are dead at the age of 64 and 66, allegedly due to kidney failure. The other affected siblings did not have children.

The proband has muscle cramps that impaired his participation in sports from about the age of 10 years. He has had CK levels up to 5000, and mostly levels have been chronically elevated but, on some occasions, normal. A muscle biopsy performed in 2004 showed some muscle necroses and many central nuclei, so unspecific myopathic. His muscle cramps have been debilitating and he received early retirement 6 years ago primarily due to his muscle symptoms. Treatment with lamotrigine for his cramps is considered.

Starting in his thirties he had visual problems, mostly in his RE. He was diagnosed with RP. He now can see only light and shades.

He developed diabetes type 2 at the age of 49 years for which he is treated with metformin only. He has elevated transaminases related to high CK. He has no other medical issues. Echocardiography and electrocardiogram were normal.

At last examination at the age of 57 years, he had a height of 1.65 m (-1.6 SD), weigh of 68 kg (+1.7 SD) and head circumference of 55 cm (-1.6 SD). Mild facial dysmorphisms were noted. Neurological exam is normal besides squints on his RE, which is almost blind.

The participant was included in the MYO-SEQ project.^5^ Exome sequencing was carried out at the Broad Institute of MIT and Harvard’s Genomics Platform (Cambridge, MA, USA) using a 38‑Mb targeted Illumina exome capture. A panel of 429 genes associated with neuromuscular conditions did not identify any relevant causative variants and the case remained unsolved. After the identification of the *POC5* variant in participant 4, a cross-cohort interrogation for putative pathogenic variants in this gene was carried out, which retrieved the nonsense *POC5* variant in this participant.

**Participant 8 – *POC5*** g.75690442_75690445del c.917_920del p.(Ala306Glufs*4) homozygous

The proband is a 35-year-old female, child from a consanguineous couple with four unaffected sisters and five brothers of which one is reportedly affected by visual impairment not further specified. She was born after an uncomplicated pregnancy at term. Her development was normal. She walked at the age of 1 year, had no delay in speech development, and followed regular education.

She experienced night blindness since the age of 10 years as well as loss of peripheral visual field and was diagnosed with RP at the age of 25 years. At first presentation, her best corrected visual acuity in the RE was 0.8 (Landolt) and 0.5 (Landolt) in the LE with a refractive error of spherical equivalent S +1.00 RE and S –1.00 LE. Slit lamp examination revealed an intraocular lens in the RE and an incipient cortico-nuclear cataract in the LE. Dilated fundus examination showed mild waxy pallor of the optic discs with some mild swelling in the LE. In the mid-periphery of both eyes a thinned retina with bone-spicule hyperpigmentation and attenuation of the vessels was visible. Optical Coherence Tomography (OCT) showed preserved outer retina foveally and parafoveally, with normal configuration of the fovea in both eyes. On fundus autofluorescence, the foveola revealed normal autofluorescence with an hyperautofluorescent ring parafoveally and hypoautofluorescent spots in the midperiphery corresponding to the bone-spicules. Electroretinography was not performed. Octopus 900 kinetic visual field showed a concentric restriction to a radius of approximately 5° using target III4e.

Through laboratory investigations, she was diagnosed with diabetes mellitus type 2 at the age of 26 years. She was also diagnosed with hepatic steatosis and renal insufficiency. Spleen and thyroid gland were unremarkable. Her hearing is normal. She had normal puberty development with a menarche at 12 years of age and has ovarian cysts.

At physical examination she had a height of 1.50 m (-2.3 SD) and a weight of 51 kg (+0.3 SD). She had acanthosis nigricans on the neck, armpit, chin, and in the inguinal area, which become darker in summer. Other abnormalities included hair loss, thin eyebrows, no eyelashes, and misalignment of deciduous teeth.

The proband received diagnostic genetic testing by disease gene panel analysis based on genome sequencing for genes known to be associated with syndromic and non-syndromic retinal disease.^6^ The initial analysis did not identify any putative causative genetic variants. Re-evaluation of the case in a research context searching for biallelic variants in *POC5* revealed a homozygous 4 bp deletion c.917_920del p.(Ala306Glufs*45) in this gene. Segregation analysis in the parents of the proband showed that both are heterozygous for the *POC5* variant.

**Participant 9 – *POC5*** g.75689120G>A c.1021C>T p.(Gln314*) homozygous

The subject is a 28-years-old female, the youngest of six children from an unaffected consanguineous couple of Afghan descent. Two sisters, living in Afghanistan, are allegedly blind and diagnosed with diabetes. One older brother is also affected with RP causing him severely decreased eyesight. He is also diagnosed with diabetes for which he is treated with vipdomet (metformin and alogliptin) and Empagliflozin. He is followed in the neurology department due to dystonic cramps.

The proband was born in Afghanistan then later raised in Denmark. As far as we know, she was born after an uncomplicated pregnancy with unknown but low body weight. Her development was regarded normal and she followed regular education. She has a small conductive unilateral hearing loss due traumatic perforation and congenital fusing of auditory ossicles. She had normal pubertal development with menarche at nine years of age. However, she had infrequent menstruations and at 16 years of age, she was diagnosed with PCOS on the basis of clinical and biochemical hyperandrogenemia and bilateral polycystic ovaries on ultrasound examination.

From her teenage years, she experienced intermittent involuntary painful muscle cramps with fixation in abnormal positions in fingers, toes, calves, flanks, and neck which were provoked by physical activity and cold weather. At age 20 years the proband was diagnosed with insulin resistant type 2 diabetes (increased C-peptide and negative GAD antibodies). She was treated with metformin, insulin, and empagliflozin. At 25 years old, she had a miscarriage in gestation week 16 of unknown cause but suspicion of unregulated diabetes. At 26 years old, she was also diagnosed with hepatic steatosis. There were no heart complaints.

Until 25 years of age, she did not have complaints about her eyesight when using correction. However, when undergoing diabetic ophthalmologic routine evaluation, further perimetry testing uncovered severe peripheral vision loss. Further ophthalmologic evaluation showed visual acuity in the RE of 1.0 Snellen with correction (S -5.25 x C. -0.50 x 109°) and in the LE 1.0 Snellen with correction (S -5.25 x C -1.50 x 011°). Slit lamp examination showed age-appropriate lenses, minimal corpus overlap, and no vitritis. Dilated fundus examination showed well-defined slightly tilted nerve heads with symmetrical temporal peripapillary atrophy and inconspicuous maculae. There was a lightly grainy pigment shift in the fundus and in the periphery thin vessels and spicule-like changes. Autofluorescence examination on OPTOS showed hyperfluorescence within the vascular arches, but with a fine distance to the fovea and speckled hypofluorescence in the peripheries. Full-field ERG showed severely reduced amplitude of scotopic and photopic A and B waves, interpreted as generalized affection of rods and cones. Visual field computometrics showed symmetrically and concentrically narrowed field of view to 15° to each side of fixation point.

At physical examination, she had a height of 1.60 m (-0.6 SD) and a weight of 41 kg (-2.2 SD, BMI 16 kg/m^2^). She had slender limbs with almost no subcutaneous fat, but presence of abdominal subcutaneous fat deposits. She also had hirsutism of the lower back.

Previous genetic testing consisting of a gene panel of genes known to be associated with maturity onset diabetes of the young type 1-13 and a gene panel of genes known to be associated with RP (including mitochondrial DNA), did not reveal an underlying cause. Trio exome sequencing was performed on the Illumina NovaSeq 6000 using Illumina® DNA PCR-Free Genome Prep. Read alignment was performed using Illumina DRAGEN Bio-IT Platform. This revealed a homozygous truncating variant introducing a premature stop codon in exon 9 of *POC5.* This variant was also identified homozygously in the affected brother.

**Participant 10 – *POC5*** g.75689087T>A c.1054A>T p.(Lys352*) homozygous

The proband is a 23-year-old female, the youngest of four children from a consanguineous couple with an unremarkable family history. She was born after an uncomplicated pregnancy at 36.5 weeks of gestation with a birth weight of 2100 gram (-2 SD). She was in an incubator for 2 weeks. She wore a Pavlik harness because of hip dysplasia during the first 3 months. She had feeding problems. Until the age of 2 years she received pureed/mashed food because of swallowing difficulties. Her development was normal. She walked at the age of 1 year, had no delay in speech development, and followed regular education. Her hearing is normal. She had normal puberty development with a menarche at 12 years of age. However, she had oligomenorrhea and her menstruations were very infrequent. She had abdominal complaints in the context of lactose intolerance. In addition, she had unexplained albuminuria.

She experienced night blindness since the age of 10 years and loss of peripheral field since the age of 19 and was diagnosed with RP. At first presentation her best corrected visual acuity in the RE was 1.0- (Snellen) and 0.9+ (Snellen) in the LE with a refractive error of spherical equivalent S -5.75 RE and S -7.50 LE.

Since the age of 15 years she developed muscle cramps in her calves. Firstly, these occurred at night, but later these occurred also during the day and extended to other muscles. She also had tingling sensations, especially in the right leg. An EMG showed no signs of a neuropathy.

Through laboratory investigations, she was diagnosed with diabetes mellitus with high insulin resistance (anti-GAD65, ANtiIA2, Zn08, and MODY negative) and low leptin (5.1 µg/L). Treatment included insulin therapy (multiple daily injections and even pump therapy), empagliflozin and metformin. She was also diagnosed with partial lipodystrophy with hypertriglyceridemia, hepatosplenomegaly and hepatic steatosis and also macroalbuminuria. She recently started treatment with recombinant human metraleptin (Myalepta^Rx^_,_ once daily subcutaneously) with good effect on hepatic steatosis (left liver lobe 15 to 10 cm, right lobe 20.5 to 17.7 cm, spleen 14 to 10.5 cm, serum triglycerides 11.15 to 1.66 mmol/mol) and glucose regulation (TIR from 32 to 72%; HbA1c 62.1 to 43.0 mmol/mol Hb). The macroalbuminuria decreased from 212.6 (albumin/creatin ratio in portion, N < 3.5) to 42.6. She now requires insulin only once a day. There were no heart complaints.

At physical examination she had a height of 1.64 m (0 SD), weight of 47 kg (-1.5 SD, BMI 17.6 kg/m^2^), and head circumference of 52.7 cm (-1.6 SD). Her scalp hair was sparse and thin and she had a high anterior hairline thick eyebrows, proptosis, prominent nose, prominent nasal tip, wide nasal base, large fleshy ears, wide mouth and a mandibular prognathia. She had a mild pectus excavatum and prominent chest veins. There was a clear loss of subcutaneous fat in the face and from the upper and lower extremities with acromegaly. Both axillary and inguinal acanthosis nigricans was observed. She had hirsutism on face, thorax, abdomen and back, and soft skin with atypical scarring. She was noted to have a deep voice.

Slit lamp examination revealed mild subcapsularis posterior cataract in both eyes. Dilated fundus examination showed mild waxy pallor of the optic disc with some mild swelling in both eyes. In the RE the macula showed mild granular changes of the RPE and attenuation of the vessels. In the mid-periphery a thinned retina with bone-spicule hyperpigmentation was visible, just peripheral from the arcades the far periphery was atrophic. In the LE a fovea reflex was present and there was narrowing of the vessels. Similar to the RE, a thinned retina with bone-spicule pigmentation was observed just peripheral from the arcades with atrophy in the far periphery.

Optical Coherence Tomography (OCT) showed preserved outer retina within the vascular arcades with normal configuration of the fovea in both eyes. On fundus autofluorescence the posterior pole showed normal autofluorescence. There was a hyperautofluorescent ring visible around the arcades and nasal of the optic nerve. This was surrounded by a ring of normal fundus autofluorescence. Peripheral from this ring was a broad ring with mottling of autofluorescence but mainly hypoautofluorescence was observed. Electroretinography revealed extinguished cone and rod responses. Octopus visual field showed a concentric restriction.

Previous genetic investigations consisting of SNP array, analysis of a panel of genes known to be associated with lipodystrophy, and metabolic screening did not reveal an underlying cause. Exome sequencing was performed on the Illumina NovaSeq 6000 after enrichment with the Twist Exome 2.0 plus Comprehensive Exome Spike-in Kit. Read alignment was performed using BWA and variant calling using GATK (SNVs) and CoNIFER and/or ExomeDepth (CNVs). This revealed a homozygous frameshift variant in *POC5*, as well as a *de novo* *ARMH1* (HGNC:34345) variant.

**Participant 11 – *POC5*** g.75689087T>A c.1054A>T p.(Lys352*) homozygous

This female is currently 58 years old. She was born in Russia and moved to Belgium in her forties with her son. She was the youngest child in a sibship of 7 from non-consanguineous parents. One sibling had renal failure. One brother, one sister and her mother had diabetes mellitus, but she was the only one with retinal dystrophy.

She was diagnosed with insulin-dependent diabetes mellitus at age 45. Arterial blood pressure and blood lipids were elevated and were satisfactorily controlled using lifestyle changes and medication, but her hyperglycemia was poorly controlled despite good efforts. She was in good cardiovascular health and had no nephropathy. Elevated γ-glutamyl transferase and mildly elevated transaminases prompted assessment of the liver including elastography which revealed advanced fibrosis and severe steatosis. A liver biopsy showed mild steatohepatitis as well as mild periportal and centrilobular fibrosis (grade F2). Endocrinological assessments excluded Cushing disease, hypogonadism and hypothyroidism. She had her first menstruation at age 13-14 and became menopausal at age 52.

She was referred to the ophthalmologist for an assessment for diabetic retinopathy at the age of 53 at which time a rod-cone dystrophy was formally diagnosed. She reported onset of vision problems during childhood, but her vision has lately been worsening markedly. She was able to read up to age 52. She reported no photophobia, nyctalopia or peripheral visual fields. Automated refraction showed S+4,00 x C-1,00 D x 109° in the RE and S+3,00 x C-0.25 x 054° in the LE. Best corrected visual acuity was at 0.5 Snellen in each eye at first assessment (age 53) and was 0.2 in each eye 2 years later. Slit-lamp exam did not show any significant anterior segment anomalies. Goldmann kinetic perimetries indicated a loss of the superior visual field with good preservation temporally, nasally and inferiorly. Pericentral sensitivity was reduced with I2e retained but smaller than the central 10° and without absolute central scotomata. Full-field electroretinography showed loss of rod-specific responses and reduced cone responses at age 53. Fundoscopy revealed outer retinal atrophy in the midperiphery over 360° and inferiorly into the far periphery with bone spicule type intraretinal pigment migration and a few punched out lesions of chorioretinal atrophy inferiorly. Short-wavelength autofluorescence corroborated the fundoscopy findings. Macular OCT showed well preserved retinal layers both in differentiation and thickness with an EZ layer that is intact apart from a small area right at the fovea.

She experiences painful muscle cramps and has chronic back pain. Musculoskeletal assessment showed degenerative joint disease of hands, feet and spine. She underwent local and surgical treatments for lumbosacral radiculopathy. She has recurrent headaches as well as painful hands, feet and eyelids. She has swelling of the face, eyelids, hands and feet and shows abdominal obesity. Her height is 1.56 m (-1.3 SD) and her weight varies between 65 and 70 kg between measurements.

Exome sequencing was performed for participant 9. Library preparation and sequencing were performed using SureSelectXT Human All Exon V7 (Agilent, CA, USA) and 150 bp paired-end sequencing (NovaSeq 6000, Illumina, CA, USA). Data were basecalled using bcl2fastq2 (v2.20) and demultiplexed with ngsutils. Basecalled reads were subsequently aligned to GRCh38 using BWA-MEM (v0.7.17), after which coverage was assessed using mosdepth (v0.3.3). Variant calling for single nucleotide variants (SNVs) was done using HaplotypeCaller from the GATK (v3.3) toolkit and annotation was performed through Ensembl Variant Effect Predictor (v110.0) and dbNSFP (v4). Post-demultiplexing steps were performed within the bcbio toolkit. Resulting SNV VCF files were analyzed using the in-house tool Seqplorer for variants in the RetNet panel (v4 , 276 genes), while copy number variations in these genes were assessed using ExomeDepth (v1.1.16). SNV analysis revealed a homozygous nonsense variant in *POC5.*

**Participant 12 – *POC5*** g.75689087dup c.1059dup p.(Ala354Serfs*12) and g.75694755_75694758del c.592_595del p.(Glu198Asnfs*6) compound heterozygous

A Spanish 41-years-old female presenting with RP, underwent comprehensive clinical evaluation, including ophthalmological examination, genetic testing, and systemic assessment. She was born at 38 weeks of gestation with a birth weight of 3300 gram and a birth length of 51 cm. No developmental abnormalities were observed.

She showed night blindness and progressive concentric restriction in visual field starting at the second decade of life. At 34 years old, her most recent ophthalmological examination revealed a concentric constriction to 15° of visual field, bone-spicule pigmentation, and vessel narrowing with preserved macula. The electroretinogram showed reduced amplitude under photopic conditions and convincingly absent responses under scotopic conditions.

Additionally, she presented with fasciculations and cramps at rest rather than during activity since infancy. She was diagnosed with myopathy around the age of 30, with electromyography revealing abnormal responses, and the muscle biopsy showing normal results. Moreover, she presented paraesthesia in her hands and feet.

She also presented with LD. The proband shows fat on upper segment, and loss fat from the upper and lower extremities, accompanied by fatty liver type 1, insulin resistance, and high antithyroid antibodies with normal thyroid function. Furthermore, she showed thin and sparse hair morphology and atopic dermatitis. The proband also exhibited micrognatia and retrognatia, late loss of baby teeth, and misalignment adult teeth, which were corrected with orthodontics. During childhood, she had scoliosis, which was corrected with a corset.

Previous genetic testing included clinical exome sequencing (Sophia Genetics) that did not reveal the cause of the IRD. Exome sequencing (WES) was performed on the Illumina NovaSeq 6000 using Twist Core Exome kit (Twist Bioscience). Bioinformatics analysis was performed using a bioinformatics pipeline previously described.^7^ Two frameshift variants in *POC5* were identified, and segregation was performed using Sanger sequencing, confirming that both variants are in trans.

1. **Results Immunofluorescence**

**
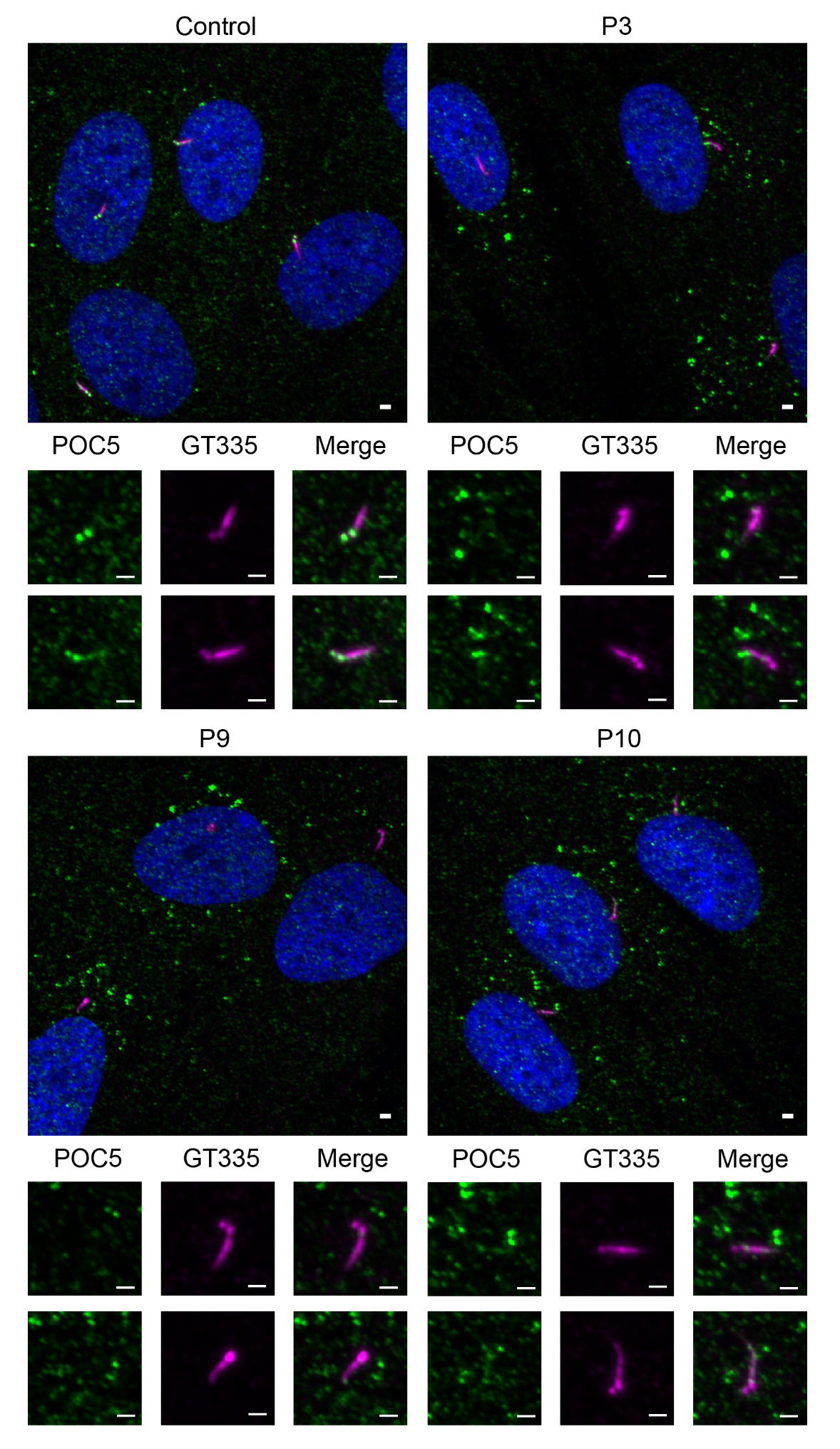
**

**Supplemental Figure 2.** Centriolar localization of POC5 in fibroblasts from a control and P3, P9 and P10. Immunofluorescent images showing centriolar POC5 localization in ciliated cells. Centrioles and the proximal end of the cilium are visualized with GT335 in purple and POC5 localization is visualized in green. Scale bar represents 1µm.

**Supplemental Table 4.**

Cilium phenotyping in fibroblasts derived from participants

| **Cell line** | **Ciliogenesis** | **Cilium length** |
| --- | --- | --- |
| Control 1 | 96±3% | 4.06±0.08µm |
| Control 2 | 90±2% | 4.32±0.12µm |
| Controls (1+2) | 92±4% | 4.19±0.17µm |
| P3 | 86±10% | 3.80±0.20µm |
| P9 | 80±14% | 4.36±0.11µm |
| P10 | 87±3% | 4.28±0.29µm |

1. **Materials and methods**

***Targeted and transcriptome-wide RNA analysis***

Targeted RNA analysis was performed on RNA from skin-derived fibroblasts from four affected participants, i.e. P3, P8, P9 and P10, and two unrelated controls. The fibroblasts were cultured in the presence or absence of cycloheximide (CHX). RNA was isolated using the Macherey-Nagel NucleoSpin RNA kit according to the manufacturers protocol. Subsequently, 500ng RNA was used to synthesize cDNA using the Maxima First Strand cDNA synthesis Kit for RT-qPCR according to the manufacturers protocol. A nearly full length POC5 transcript was amplified with PCR using a forward primer located in exon 2 and a reverse primer in exon 12 (See supplementary table 2 for primer sequences). Transcriptome-wide analysis was performed for P3 as described by Dekker *et al*..^2^ In brief, RNA was isolated from fibroblasts cultured with and without CHX. mRNA enriched total RNA was sequenced on an Illumina NovaSeq 6000 with 150 bp paired-end reads. FASTQ files were processed with an established pipeline at the Department of Clinical Genetics Erasmus MC (Rotterdam, the Netherlands).^2^ Expression outliers were picked-up by a transcriptome-analysis web application identifying abnormal transcripts in one dataset compared to a control cohort using exon, intron and gene level outliers based on the OUTRIDER algorithm.^8^

***Quantitative real-time polymerase chain reaction (qRT-PCR)***

Total RNA was extracted from fibroblasts from P3, P8, P9 and P10 using the NucleoSpin RNA (Macherey-Nagel GmbH & Co. KG, Düren, Germany). Sample concentration and purity values were measured using Nanodrop spectrophotometer (Thermo Fisher Scientific, Waltham, MA, USA). cDNA was synthesized using the iScript cDNA synthesis kit (Bio-Rad, Hercules, CA, USA) according to the manufacturer’s instructions. The relative expression levels of *POC5* were determined by quantitative RT-PCR using GoTaq qPCR Master mix (Promega, Madison, WI, USA). The results were analyzed using the Delta-delta-Ct method, using *GUSB* as a housekeeping gene for normalization. *POC5* expression levels were calculated relative to the controls for the treated and untreated samples. The primers used are listed in the Supplementary Table 3.

***Immunofluorescence***

Fibroblasts derived from participants 3, 8, 9 and 10 were cultured in Dulbecco’s modified Eagle’s medium (DMEM) with 20% FCS on sterile coverslips to approximately 90% confluence. To stimulate cilium formation, the cells were starved for 48 hours using DMEM with 0.2% FCS. For the SHH assay the cells were stimulated with 500 nM smoothened agonist for 24 hours prior to fixation. A standard immunofluorescence protocol was used.^9^ In brief, the cells were fixated in 2% paraformaldehyde in PBS for 20 min, permeabilized with 1% Triton-X-100 in PBS for 5 min and blocked for 30 min with 2% bovine serum albumin (BSA) in PBS. Subsequently, the primary antibody and secondary antibody were added to the cells for 1 hr at room temperature with a thorough wash in between the incubations with antibodies. The following primary antibodies were used: anti-POC5 (rabbit polyclonal, cat#HPA037510, Sigma-Aldrich, Zwijndrecht, Netherlands, 1:250), anti-ARL13B (rabbit polyclonal, cat#17711-1-AP, Proteintech Group, Manchester, UK, 1:100), anti-IFT88 (rabbit polyclonal, cat#13967-1-AP, Proteintech Group, Manchester, United Kingdom, 1:100), anti-acetylated-α-tubulin (mouse monoclonal, cat#T6793, Sigma-Aldrich, Zwijndrecht, Netherlands, 1:1000), GT335 (mouse monoclonal, gift from Dr. C. Janke, CNRS Centre de Recherches en Biochimie Macromoléculaire, Montpellier, France), anti-centrin (mouse monoclonal, cat#04-1624, Sigma-Aldrich, Zwijndrecht, Netherlands, 1:1000) anti-pericentrin (PCNT, mouse monoclonal, cat#ab28144, Abcam, Cambridge, United Kingdom, 1:1000), smoothened (mouse monoclonal, cat#sc-166685, Santa Cruz, 1:500) and GPR161 (rabbit polyclonal, Cat#13398-1, Proteintech Group, Manchester, United Kingdom, 1:1000), in combination with the following secondary antibodies: anti-rabbit Alexa Fluor 488, anti-mouse IgG2B Alexa Fluor 568, anti-mouse IgG2A Alexa Fluor 568 and anti-mouse IgG1 Alexa Fluor 647, obtained from ThermoFischer Scientific Waltham, USA. Finally, the coverslips were embedded in Fluoromount-G with DAPI (Southern Biotech, Birmingham, AL, USA) on a microscopic glass slide. The coverslips were analyzed with a Zeiss Axio Imager Z2 microscope (Zeiss, Sliedrecht, Netherlands) equipped with an ApoTome slider. The experiments were performed in triplicate. The ciliary phenotype, including ciliogenesis and cilium length, was determined in an automated manner using ALPACA as described by Doornbos et al.^9^

1. **References**

1. Weisz Hubshman M, Broekman S, van Wijk E, et al. Whole-exome sequencing reveals POC5 as a novel gene associated with autosomal recessive retinitis pigmentosa. *Hum Mol Genet*. 2018;27(4):614-24. <https://www.ncbi.nlm.nih.gov/pubmed/29272404>

2. Dekker J, Schot R, Bongaerts M, et al. Web-accessible application for identifying pathogenic transcripts with RNA-seq: Increased sensitivity in diagnosis of neurodevelopmental disorders. *Am J Hum Genet*. 2023;110(2):251-72. <https://www.ncbi.nlm.nih.gov/pubmed/36669495>

3. Prasun P, LoPiccolo MK, Ginevic I. Long-Chain Hydroxyacyl-CoA Dehydrogenase Deficiency / Trifunctional Protein Deficiency. In: Adam MP, Feldman J, Mirzaa GM, Pagon RA, Wallace SE, Amemiya A, editors. GeneReviews((R)). Seattle (WA)1993.

4. Martin AR, Williams E, Foulger RE, et al. PanelApp crowdsources expert knowledge to establish consensus diagnostic gene panels. *Nat Genet*. 2019;51(11):1560-5. <https://www.ncbi.nlm.nih.gov/pubmed/31676867>

5. Topf A, Johnson K, Bates A, et al. Sequential targeted exome sequencing of 1001 patients affected by unexplained limb-girdle weakness. *Genet Med*. 2020;22(9):1478-88. <https://www.ncbi.nlm.nih.gov/pubmed/32528171>

6. Weisschuh N, Mazzola P, Zuleger T, et al. Diagnostic genome sequencing improves diagnostic yield: a prospective single-centre study in 1000 patients with inherited eye diseases. *J Med Genet*. 2024;61(2):186-95. <https://www.ncbi.nlm.nih.gov/pubmed/37734845>

7. Peter VG, Kaminska K, Santos C, et al. The first genetic landscape of inherited retinal dystrophies in Portuguese patients identifies recurrent homozygous mutations as a frequent cause of pathogenesis. *PNAS Nexus*. 2023;2(3):pgad043. <https://www.ncbi.nlm.nih.gov/pubmed/36909829>

8. Brechtmann F, Mertes C, Matuseviciute A, et al. OUTRIDER: A Statistical Method for Detecting Aberrantly Expressed Genes in RNA Sequencing Data. *Am J Hum Genet*. 2018;103(6):907-17. <https://www.ncbi.nlm.nih.gov/pubmed/30503520>

9. Doornbos C, van Beek R, Bongers E, et al. Cell-based assay for ciliopathy patients to improve accurate diagnosis using ALPACA. *Eur J Hum Genet*. 2021;29(11):1677-89. <https://www.ncbi.nlm.nih.gov/pubmed/34040173>
